# The role of maternal-specific H3K9me3 modification in establishing imprinted X-chromosome inactivation and embryogenesis in mice

**DOI:** 10.1038/ncomms6464

**Published:** 2014-11-14

**Authors:** Atsushi Fukuda, Junko Tomikawa, Takumi Miura, Kenichiro Hata, Kazuhiko Nakabayashi, Kevin Eggan, Hidenori Akutsu, Akihiro Umezawa

**Affiliations:** 1 Department of Reproductive Biology, National Research Institute for Child Health and Development, 2-10-1 Okura, Setagaya, Tokyo 157-8535, Japan; 2 Department of Maternal-Foetal Biology, National Research Institute for Child Health and Development, 2-10-1 Okura, Setagaya, Tokyo 157-8535, Japan; 3 The Howard Hughes Medical Institute, Harvard Stem Cell Institute and the Department of Stem Cell and Regenerative Biology, Harvard University, 7 Divinity Avenue, Cambridge, Massachusetts 02138, USA

## Abstract

Maintaining a single active X-chromosome by repressing *Xist* is crucial for embryonic development in mice. Although the *Xist* activator RNF12/RLIM is present as a maternal factor, maternal *Xist* (Xm-*Xist*) is repressed during preimplantation phases to establish imprinted X-chromosome inactivation (XCI). Here we show, using a highly reproducible chromatin immunoprecipitation method that facilitates chromatin analysis of preimplantation embryos, that H3K9me3 is enriched at the *Xist* promoter region, preventing Xm-*Xist* activation by RNF12. The high levels of H3K9me3 at the *Xist* promoter region are lost in embryonic stem (ES) cells, and ES-cloned embryos show RNF12-dependent *Xist* expression. Moreover, lack of Xm-XCI in the trophectoderm, rather than loss of paternally expressed imprinted genes, is the primary cause of embryonic lethality in 70–80% of parthenogenotes immediately after implantation. This study reveals that H3K9me3 is involved in the imprinting that silences Xm-*Xist*. Our findings highlight the role of maternal-specific H3K9me3 modification in embryo development.

## Abstract (web summary)

During mouse preimplantation phases, a repressive imprint is imposed on the maternal allele of *Xist*, which encodes a large non-coding RNA required for X-chromosome inactivation. Here the authors show that trimethylation of histone H3 at lysine 9 on *Xist* promoter chromatin is responsible for the maternally determined *Xist* repression.

## Graphical abstract

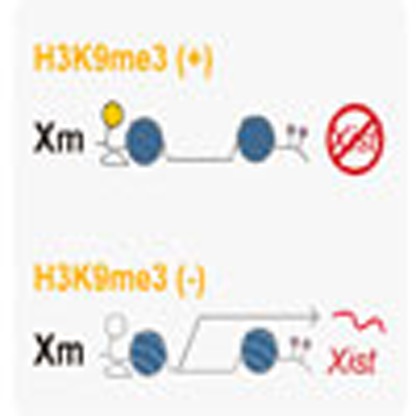

To maintain proper dosage compensation in mammals, one of the two X chromosomes in the female is inactivated^1^,^2^. In establishment of X-chromosome inactivation (XCI), a large non-coding RNA, *Xist*, is expressed and this non-coding RNA then covers the entire X chromosome in *cis*^1^,^2^,^3^. In mice, two types of XCI occur during female embryonic development. One type involves random XCI, which is observed in cells derived from epiblasts, and one of the two X chromosomes (paternal or maternal) is randomly inactivated. The other involves imprinted XCI (iXCI), which is observed in extra-embryonic tissues and causes XCI of the paternal X chromosome (Xp)^4^. The initiation of iXCI begins at early preimplantation in embryos and Xp-*Xist* is expressed around the four-cell stage^1^. A recent study showed that a maternal factor, the E3 ubiquitin ligase RNF12, is the primary factor responsible for Xp-*Xist* activation^5^. Interestingly, although RNF12 is abundant as a maternal factor in oocytes, Xm-*Xist* is not expressed. Moreover, maternal *Xist* (Xm-*Xist*)-specific imprints, which are refractory to the Xm-*Xist* activation induced by RNF12, are imposed during oogenesis^6^. *Xist* expression analysis using *de novo* DNA methyltransferase (*Dnmt3a*/*b*) maternal knockout mice demonstrated that *Xist* expression during preimplantation is independent of DNA methylation^7^, implying that other epigenetic factors are associated with Xm-*Xist* silencing. However, the nature of these Xm-specific epigenetic modifications is unknown.

A gene-knockout study demonstrated that loss of Xp*-Xist* expression critically affects postimplantation female development due to lack of iXCI, which causes overexpression of X-linked genes in extra-embryonic tissues^8^. Similar to the phenotype observed in Xp-*Xist*-knockout mice, parthenogenetic embryos (PEs) composed of two X chromosomes show increased expression of X-linked genes, as compared with fertilized females, because of the low expression of *Xist*^9^. One of the interesting phenomena observed in PEs is the dramatic developmental failure that occurs immediately after implantation. Around 70–80% of embryos die before embryonic day (E) 9.5, which is the limit of development for PEs^10^. However, it is unknown whether the primary cause of rapid developmental failure in postimplantation PEs is the loss of iXCI or the loss of expression of autosomal paternally imprinted genes^11^,^12^.

The global epigenetic asymmetry of parental genomes in zygotes is retained during early preimplantation phases in mice and changes in gene expression occur in discrete stages to confer totipotency^13^,^14^. Interestingly, transcriptionally repressive marks, such as histone H3 lysine 9 di-/trimethylation (H3K9me2/3), are specifically imposed on maternal genomes at the zygote stage^13^. Although the regulation of imprinted genes mostly depends on DNA methylation, some imprinted genes are regulated by these histone modifications^15^,^16^. Thus, Xm-*Xist* silencing machinery may be associated with histone modifications.

Here we reveal that silencing of Xm-*Xist* in preimplantation embryos involves modification of H3K9me3. By using a new chromatin immunoprecipitation (ChIP) method that facilitates chromatin analysis in preimplantation embryos, we show that the *Xist* promoter on the Xm is highly enriched for H3K9me3 at the four-cell stage. This enrichment is lost in the morula and in male embryonic stem (ES) cells. Furthermore, we demonstrate that early loss of H3K9me3 at the *Xist* promoter leads to precocious Xm-*Xist* activation in a Rnf12-dependent manner. Moreover, we demonstrate that establishment of Xm-XCI in the trophectoderm allows PEs to develop at the postimplantation stage without the expression of paternally imprinted genes on autosomes. Therefore, these data indicate that the primary cause of embryonic lethality immediately after implantation in most PEs is loss of XCI rather than loss of the expression of paternally imprinted genes located on autosomes. Our study revealed that silencing of Xm-*Xist* by imprinting to establish iXCI involves H3K9me3, and this finding is expected to resolve the longstanding issues that have limited our general understanding of XCI in mice.

## Results

### Changes in histone modifications cause Xm-*Xist* derepression

**Figure 1:**
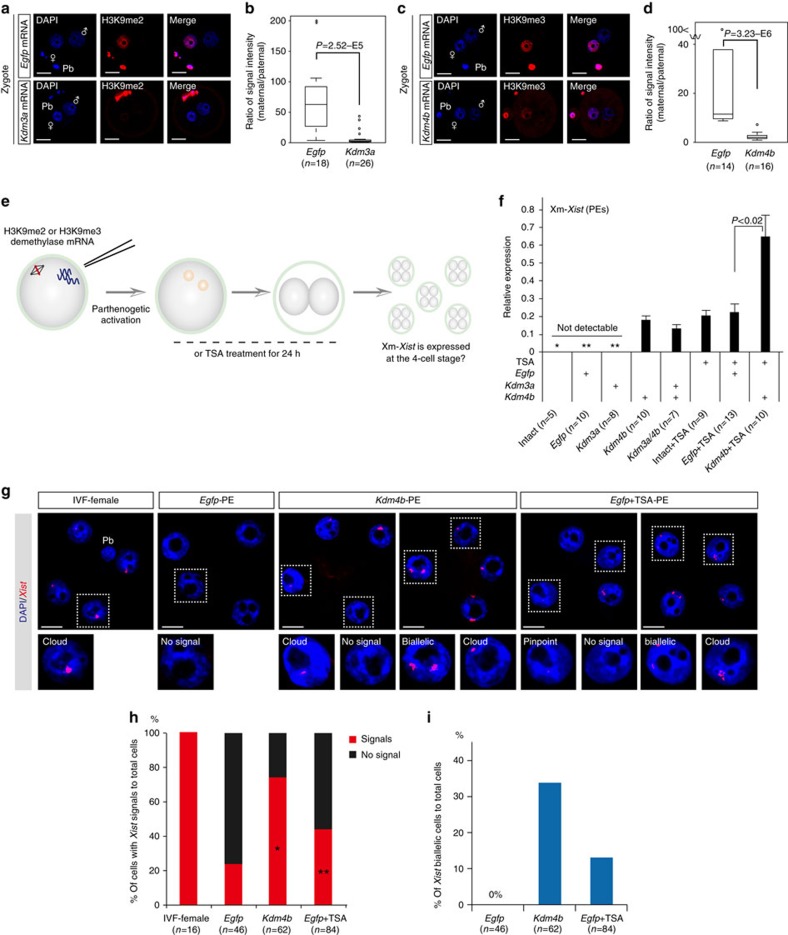
Alterations in histone modifications derepress Xm-*Xist* expression. (**a**–**d**) Oocytes injected with *Kdm3a* (**a**,**b**), *Kdm4b* (**c**,**d**) or *Egfp* mRNAs were subjected to ICSI. After 7–8 h, embryos were fixed and analysed for H3K9me2 (**a**) and H3K9me3 (**c**) using IF. Nuclei stained with 4′,6-diamidino-2-phenylindole (DAPI) are shown in blue. Representative images are presented on the left. The box-and-whisker plot shows the ratio of maternal to paternal signal intensities. The horizontal line indicates the median. The *P*-values were calculated using the Mann–Whitney *U*-test (*U*-test). Pb, polar body; *n*, number of embryos analysed (**b**,**d**). (**e**) Schema of the generation of PEs with altered histone modifications. To examine the effects of histone demethylation on Xm-*Xist* expression, either H3K9me2 demethylase (*Kdm3a*) or H3K9me3 demethylase (*Kdm4b*) mRNAs were injected into MII oocytes that were then activated. To assess the effects of inhibition of histone deacetylation on Xm-*Xist* expression, oocytes were activated and incubated in the presence of TSA for 24 h. After 48 h, ten four-cell PEs were pooled and analysed as one biological replicate using qPCR. (**f**) Analysis of Xm-*Xist* expression at the four-cell stage. The expression level of Xm-*Xist* in female embryos derived from IVF was defined as 1. One or two asterisks indicate Xm-*Xist* expression in one or two replicates, respectively. The *P*-values were determined using Student’s *t*-tests. Error bars indicate the mean±s.e.m. (**g**–**i**) *Xist* FISH analysis of *Kdm4b*- and *Egfp*+TSA-PEs at the four-cell stage. (**g**) Representative images of FISH results. (**h**) Ratio of cells with *Xist* signal to the total number of cells. *n*, number of interphase cells analysed. (**i**) Ratio of cells with biallelic expression to total cells. The detailed FISH results are shown in Supplementary Table 1. Scale bars, 20 μm.

Histone repressive marks, such as H3K9me2/3 and H3K27me3, are specifically imposed on maternal genomes^13^. To investigate the role of maternal-specific modifications in imprinted *Xist* expression, we focused on *Kdm3a* and *Kdm4b*, which encode histone demethylases specific for H3K9me1/2 and H3K9me3 (refs 17, 18), respectively. Reverse transcription–PCR analysis showed that oocytes express low levels of *Kdm3a* and *Kdm4b* (Supplementary Fig. 1). Immunofluorescence (IF) analyses revealed that zygotes injected with polyadenylated *Kdm3a* and *Kdm4b* messenger RNAs expressed significantly lower levels of maternal H3K9me2 and H3K9me3, respectively (Fig. 1a–d). Ectopic expression of *Kdm3a* and *Kdm4b* did not affect H3K9me3 or H3K9me2 marks, respectively (Supplementary Fig. 2). We reasoned that if Xm-specific modifications that prevent *Xist* activation were erased by these epigenetic modifiers, Xm-*Xist* would be expressed at the four-cell stage, which is when Xp-*Xist* expression commences.

To facilitate analysis of Xm-*Xist* expression, we used PEs (Fig. 1e). PEs possess two copies of Xm, and Xm-*Xist* is never expressed at the four-cell stage^19^. Xm-*Xist* expression in four-cell PEs, cultured for 48 h, was determined using quantitative real-time PCR (qPCR). Consistent with a previous report^19^, Xm-*Xist* was not detectably expressed in most intact (not injected) PEs and PEs injected with *Egfp* mRNA (*Egfp*-PEs; Fig. 1f). Approximately 75% of PEs injected with *Kdm3a* mRNA (*Kdm3a*-PEs) did not detectably express *Xist*. However, Xm-*Xist* expression was detected in all PEs injected with *Kdm4b* mRNA (*Kdm4b*-PEs; Fig. 1f), suggesting that H3K9me3 demethylation caused Xm-*Xist* derepression.

We next assessed the effects of a histone deacetylase inhibitor, trichostatin A (TSA), on Xm-*Xist* expression. TSA-treated PEs (Intact+TSA-PEs and *Egfp*+TSA-PEs) also activated Xm-*Xist* (Fig. 1f). No significant changes were detected in Xm-*Xist* expression levels between *Kdm4b*-PEs and *Egfp*+TSA-PEs. However, although co-injection with *Kdm4b* and *Kdm3a* mRNAs did not increase Xm-*Xist* expression levels as compared with *Kdm4b*-PEs, a combination of TSA and *Kdm4b*-mRNA significantly increased Xm-*Xist* expression as compared with *Egfp*+TSA-PEs (2.9-fold, *P*<0.04, Student’s *t*-test; Fig. 1f). Moreover, derepression of Xm-*Xist* transcription occurred in the absence of *Rnf12* overexpression (Supplementary Fig. 3), and *Jpx* and *Ftx*, which have been identified as *Xist* activators^2^, were not expressed at the four-cell stage. These results showed that KDM4B- and TSA-mediated Xm-*Xist* derepression was not involved in the abnormal expression of known *Xist* activators.

Next, we examined Xm-*Xist* derepression states at the single-cell level by fluorescence *in situ* hybridisation (FISH) of *Xist* RNA. Consistent with the qPCR results, Xm-*Xist* signals were significantly increased in *Egfp*+TSA- and *Kdm4b*-PEs (Fig. 1g,h and Supplementary Table 1). However, neither TSA treatment nor *Kdm4b* overexpression consistently activated Xm-*Xist* in all cells (Fig. 1g,h). FISH analysis also revealed *Xist* biallelic cells in *Kdm4b*- and *Egfp*+TSA-PEs (*Kdm4b*-PEs: 34% and *Egfp*+TSA-PEs: 13%; Fig. 1g,i). Taken together, these results indicated that *Kdm4b* overexpression and TSA treatment induced Xm-*Xist* derepression at the same developmental stage as Xp-*Xist* activation.

### Xm-*Xist* transcripts establish XCI at the blastocyst stage

**Figure 2:**
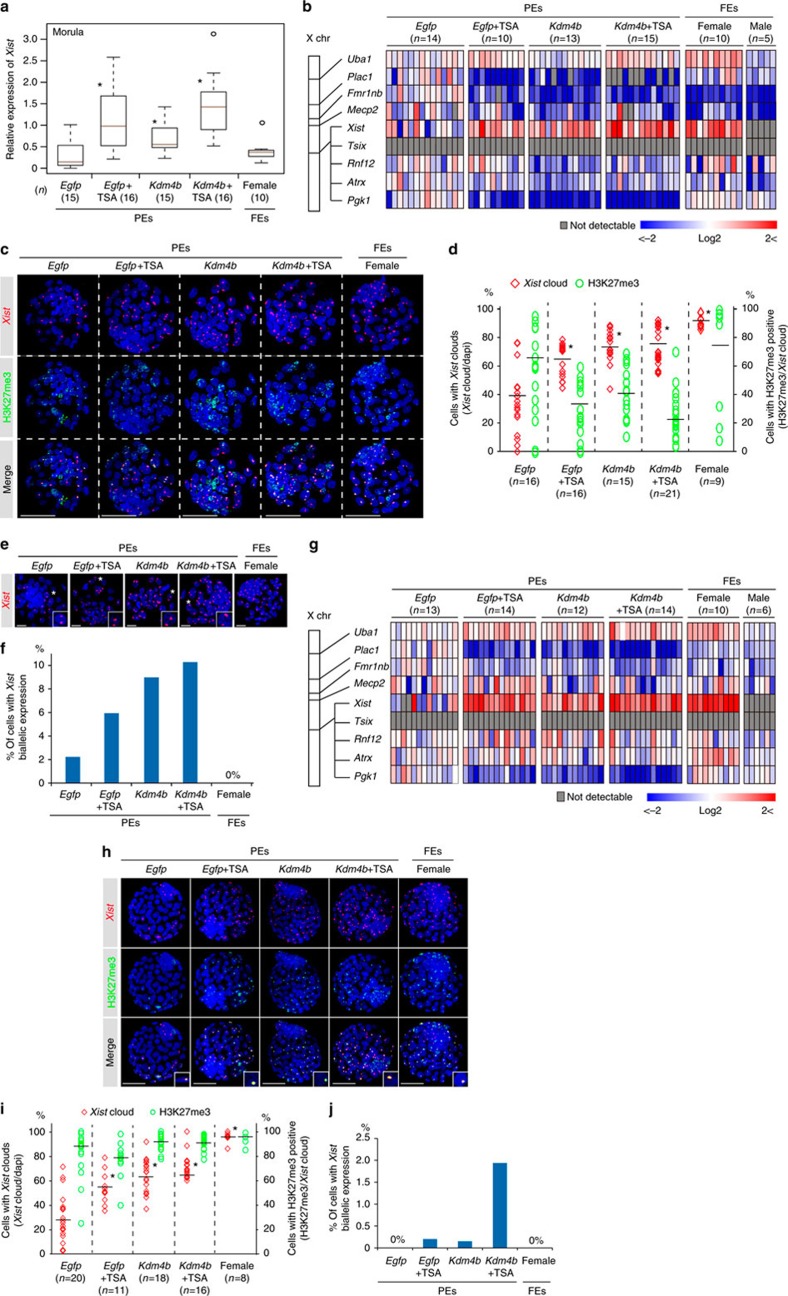
Global XCI and *Xist* expression states of Xm at late preimplantation stages. (**a**) Analysis of *Xist* expression using qPCR of individual embryos in the morula. An asterisk indicates *P*<0.05 (Student’s *t*-test) compared with *Egfp*-PEs. FEs, fertilized embryos. (**b**) Large-scale qPCR analysis of *Xist* and eight X-linked genes in individual blastocysts after culturing for 96 h. Coloured bars indicate expression levels. (**c**,**d**) IF (H3K27me3, green) combined with RNA FISH (*Xist*, red) analysis in 96-h blastocyst stage. 4′,6-diamidino-2-phenylindole (DAPI)-stained nuclei are shown in blue. (**c**) Representative confocal projection. Scale bars, 50 μm. (**d**) The graph shows *Xist* expression and H3K27me3 modification states in individual embryos. The horizontal axis indicates the average percentage in the group. *P<3.1 × 10^−28^ (Fisher’s exact test). *n*, number of embryos analysed. (**e**,**f**) Xm-*Xist* biallelic expression states in PEs at 96 h. The asterisk indicates cells with biallelic expression. Scale bars, 20 μm. (**f**) Summary of the ratio of biallelic cells to *Xist*-positive cells in 96-h blastocyst stage in each group. The number of cells is shown in Supplementary Table 3. (**g**) qPCR analysis of *Xist* and eight X-linked genes in individual blastocysts after culturing for 120 h. (**h**,**i**) IF (H3K27me3, green) combined with RNA FISH (*Xist*, red) analysis in 120 h blastocysts. *P<5.4 × 10^−23^ (Fisher’s exact test). Scale bars, 50 μm. (**j**) The ratio of biallelic cells to *Xist*-positive cells in 120 h blastocysts. In qPCR analysis, the average expression level of Xm-*Xist* in *Egfp*-PEs was set as 1 (also see the Methods section). *Gapdh* and *β-actin* were used as internal controls. Data are summarized in Supplementary Table 4.

To investigate whether Xm-*Xist* transcripts from the four-cell stage induced XCI in late preimplantation stages, we cultured *Kdm4b*-, *Egfp*+TSA- and *Kdm4b*+TSA-PEs until the blastocyst stage. Development into blastocysts occurred in >80% of PEs in all groups (Supplementary Table 2). At the morula stage (72 h), although *Egfp*-PEs initiated Xm-*Xist* expression, the levels of *Xist* expression were significantly lower compared with those in *Kdm4b*-injected and/or TSA-treated PEs (Fig. 2a). At the 96-h blastocyst stage, we examined the expression levels of *Xist* and X-linked genes (*Tsix*, *Rnf12*, *Pgk1*, *Fmr1nb*, *Atrx*, *Uba1*, *Mecp2* and *Plac1*) in individual embryos. The significant upregulation of *Xist* observed in PEs that had been injected with *Kdm4b* mRNA and/or TSA continued in 96-h blastocyst stage (Fig. 2b). In PEs exhibiting Xm-*Xist* upregulation, *Tsix* expression was not detectable and *Rnf12* was not overexpressed as compared with *Egfp*-PEs (Fig. 2b). The average expression levels of *Pgk1*, *Plac1* and *Fmr1nb* in *Kdm4b*-overexpressing or TSA-treated PE groups were significantly reduced (Supplementary Fig. 4a). However, single embryos in the same group exhibited heterogeneity in the expression levels of these targets (Fig. 2b). Moreover, although Xm-*Xist* was not overexpressed in *Kdm4b*-overexpressing or TSA-treated PE groups compared with levels seen in female fertilized embryos (FEs), the levels of *Plac1* and *Pgk1* were strongly downregulated in *Kdm4b*-, *Egfp*+TSA- and *Kdm4b*+TSA groups (Fig. 2b). These results suggested that Xm-*Xist* expression states differed at the single-cell level in individual embryos.

To gain further insights into *Xist* expression states and repression of X-linked genes on Xm alleles, we conducted IF to detect the H3K27me3 state, which is a hallmark of XCI^20^, and performed *Xist* FISH analysis. The *Xist* RNA FISH probe recognizes *Xist* and *Tsix*. Therefore, the cloud state of the FISH signal defined *Xist* expression. Consistent with the qPCR results, the number of cells with *Xist* signals increased significantly in all *Kdm4b*-overexpressing or TSA-treated PEs (Fig. 2c and Supplementary Table 3). However, in all *Kdm4b*-overexpressing or TSA-treated PEs, <45% of *Xist* cloud-containing cells had an H3K27me3 signal (Fig. 2c,d and Supplementary Table 3). We also found that *Xist* biallelic cells were present in all PE groups (*Egfp*-PEs: 2.2%, *Egfp*+TSA-PEs: 5.9%, *Kdm4b*-PEs: 9.0% and *Kdm4b*+TSA-PEs: 10.3%; Fig. 2e,f). These results suggested that there were various Xm-*Xist* expression states present at the single-cell level, affecting the heterogeneity of X-linked genes, and that strong suppression of some X-linked genes in *Kdm4b*-overexpressing or TSA-treated PEs could be attributed to biallelic expression of Xm-*Xist*.

As *Kdm4b* overexpression and TSA treatment did not affect the extent of H3K27me3 modification (Supplementary Fig. 5), its acquisition in PEs may have been slightly delayed compared with that in FEs. In support of this notion, the developmental timing of PEs lags behind that of FEs^9^. Therefore, we extended the culture period to 120 h and again performed qPCR, IF and FISH analyses. As seen in 96-h blastocyst stage, qPCR analysis revealed that *Plac1* and *Pgk1* were significantly repressed in *Kdm4b*-overexpressing or TSA-treated PEs, although heterogeneity was observed (Fig. 2g and Supplementary Fig. 4b). However, *Fmr1nb*, which was significantly repressed in 96-h blastocyst stage of both *Kdm4b*- and TSA-treated groups, did not show marked suppression (Fig. 2g and Supplementary Fig. 4b), suggesting that *Xist* expression states were altered in 120 h blastocysts.

In FISH and IF analysis, a significant increase in the number of *Xist*-positive cells was detected in *Kdm4b*-overexpressing and/or TSA-treated PEs, as compared with *Egfp*-PEs, after culture for 120 h (Fig. 2h,i and Supplementary Table 4). There was a marked increase in the ratio of H3K27me3 spots to cloud *Xist* signals in blastocysts in all groups compared with that observed after 96 h of culture as follows: 88.1% in *Egfp*-PEs, 78.7% in *Egfp*+TSA-PEs, 91.8% in *Kdm4b*-PEs and 91.2% in *Kdm4b*+TSA-PEs (Fig. 2h,i and Supplementary Table 4). Interestingly, >98% of the cells in the *Xist* cloud state exhibited monoallelic *Xist* expression in all groups at 120 h (Fig. 2j).

Taken together, these results indicated that *Kdm4b* overexpression and TSA treatment induced global XCI of Xm in blastocysts and that the counting mechanism automatically functioned in late blastocysts, as has also been observed for Xp alleles^21^.

### KDM4B-mediated Xm*-Xist* expression depends on RNF12

**Figure 3:**
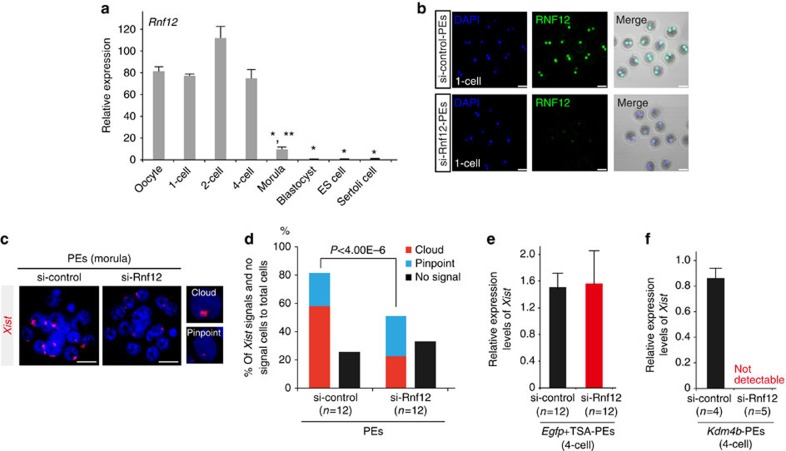
RNF12 is required for *Xist* expression in various types of preimplantation embryos. (**a**) *Rnf12* expression profiles in preimplantation stages, ES cells and somatic cells. Ten oocytes (*n*=3), ten IVF-1 cells (*n*=3), ten IVF-2 cells (*n*=3) and five IVF-4 cells (*n*=3) were pooled. The expression level of *Rnf12* in the morula stage, as determined using qPCR, represents the average of 16 individual embryos. The numbers of ES and Sertoli cells represent the averages of three independent cell lines and male pups, respectively. The error bars indicate the mean±s.e.m. *P<0.004 compared with oocytes; **P<0.0001 compared with ES cells (Student’s *t*-test). (**b**) IF analysis of RNF12 at the one-cell stage (green). Samples were fixed 11 h after parthenogenetic activation (18–19 h after siRNA injection). The same laser beam intensities were used to excite the *Rnf12*-knockdown and control samples. 4′,6-diamidino-2-phenylindole (DAPI)-stained nuclei are shown in blue. Two independent experiments were conducted. Scale bars, 50 μm. (**c**,**d**) *Xist* FISH analysis of si-Rnf12 PEs at the morula stage. At 72 h after activation, PEs injected with siRNA were analysed. Representative images of siRNA-treated embryos (**c**). Scale bars, 20 μm. The percentage of total *Xist*-positive signals and -negative cells to total cells in si-Rnf12 and si-control PEs. Biallelic expression was counted as two signals. *n*, number of embryos analysed (**d**). (**e**,**f**) qPCR analysis of Xm-*Xist* expression at the four-cell stage of embryos treated with TSA (**e**) or injected with *Kdm4b* mRNA (**f**). PEs derived from maternal si-Rnf12-treated oocytes. A detailed experimental scheme is shown in Supplementary Fig. 6d. A pool of eight to ten four-cell embryos represents one biological replicate.

During preimplantation phases, Xp-*Xist* expression is induced by maternal RNF12 (ref. 5). Thus, we investigated whether Xm-*Xist* expression also depended on RNF12. High *Rnf12* expression levels were maintained until the four-cell stage (around 80-fold higher than in ES cells; Fig. 3a). At the morula stage, although the expression level was significantly decreased compared with that in oocytes, *Rnf12* expression was still more than ninefold higher than that in ES cells (Fig. 3a), indicating that maternal and early zygotic RNF12 may be critical for Xm-*Xist* activation. To examine the dependency of RNF12 on Xm-*Xist* expression, we inhibited maternal and zygotic RNF12 expression using *Rnf12*-siRNA (Supplementary Fig. 6a). IF analysis at the one-cell stage showed a marked decline in RNF12 signal intensity in si-*Rnf12* embryos compared with that in the si-control embryos (Fig. 3b). Significant repression by si-*Rnf12* was maintained in the four-cell stage (Supplementary Fig. 6b,c). Using this knockdown system, we examined whether Xm-*Xist* activation was induced by RNF12 at the morula stage. *Xist* FISH analysis revealed that *Xist* signals (cloud and pinpoint) in PEs treated with si-Rnf12 were significantly reduced at the morula stage as compared with those observed in the controls (si-Rnf12: 50.0% versus si-control: 80.0%; Fig. 3c,d and Supplementary Table 5).

Next, we examined whether Xm-*Xist* derepression from the four-cell stage by ectopic *Kdm4b* expression or TSA treatment was regulated by RNF12 (Supplementary Fig. 6d). Four-cell embryos of *Kdm4b*- or *Egfp*+TSA-PEs treated with si-Rnf12 were analysed by qPCR. Depletion of RNF12 did not affect Xm-*Xist* expression in *Egfp*+TSA-PEs compared with the si-control PEs (Fig. 3e), suggesting that factors other than RNF12 may contribute to histone acetylation-mediated Xm-*Xist* activation. This observation is consistent with results obtained using *Rnf12*^*−/−*^ mice, which demonstrated that *Xist* is activated by RNF12 and other unidentified factors^5^,^22^. In contrast, *Xist* expression in *Kdm4b*-PEs derived from oocytes treated with si-*Rnf12* did not induce detectable expression of Xm-*Xist* (Fig. 3f). These results demonstrated that KDM4B-mediated Xm-*Xist* expression depended on RNF12 and suggested that H3K9me3 prevented the expression.

### Promoter demethylation of H3K9me3 causes Xm-*Xist* derepression

**Figure 4:**
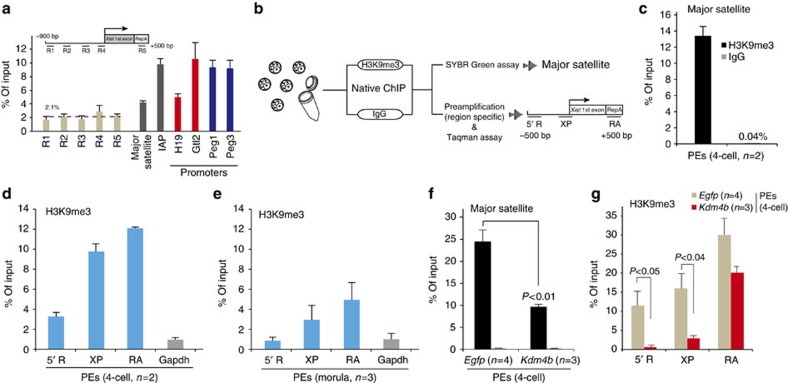
H3K9me3 states at the Xm-*Xist* promoter region in preimplantation embryos. (**a**) ChIP-qPCR analysis of Xp (sperm) at *Xist* 5′-regions, the *H19*, *Gtl2*, *Peg1* and *Peg3* promoter region, and regions in repetitive elements. *n*=2–4. Rabbit IgG was used as a negative control. The percentage of input for negative control DNA was >1% for all genes tested. The data were not normalized for local nucleosome occupancies. (**b**) Schematic representation of eChIP-qPCR analysis. H3K9me3 states at major satellite regions (**c**) and at *Xist* regions and the *Gapdh* promoter region (**d**) in PEs at the four-cell stage. Two independent experiments were performed. In each experiment, 250 embryos were used. (**e**) H3K9me3 states at the *Xist* and *Gapdh* promoter regions in morula-stage embryos. Three independent experiments were conducted and 40 embryos were used for each assay. H3K9me3 states in major satellite (**f**) and *Xist* regions (**g**) in *Egfp*- and *Kdm4b*-PEs at the four-cell stage. Three (*Kdm4b*-PEs) and four (*Egfp*-PEs) independent experiments were conducted. In each experiment, 170–250 embryos were prepared. The percentages of input for negative controls (IgG) were <0.2% (**f**) and 1.9% (**g**), respectively. Error bars indicate the mean±s.e.m. The *P*-values were determined using Student’s *t*-tests.

As activation of *Xist* by RNF12 is essential for establishing iXCI^5^, we attempted to determine the mechanism responsible for the transcriptional derepression of Xm-*Xist* by KDM4B-mediated demethylation of H3K9me3. We first examined whether H3K9me3 levels were enriched at the Xp-*Xist* promoter region. Nucleosomes were extracted from the sperm genome (Supplementary Fig. 7). ChIP-qPCR revealed the low H3K9me3 levels of Xp-*Xist* in the 5′-regions containing the major promoter for *Xist* expression (average: 2.1%; Fig. 4a), as compared with those of the *H19*, *Gtl2*, *Peg1* and *Peg3* promoter loci (average: 8.5%) and regions in repetitive elements (intracisternal A-particles and major satellite DNAs; average: 7.0%), which are known to be associated with H3K9me3 (ref. 23). These results indicated that the *Xist* promoter region was hypomethylated in sperm, in agreement with Xp-*Xist* being expressed in early embryogenesis.

Preparing sufficient numbers of embryos or oocytes for ChIP combined with deep sequencing (ChIP-seq) analysis is problematic. Some ChIP-qPCR methods requiring small samples have been reported^24^; however, most of these studies are based on a cross-linking ChIP method, in which the ChIP efficiency is lower than that of native ChIP methods^25^. Thus, we developed a new native ChIP method combined with a Taqman qPCR system for quantification of transcription in single cells (termed eChIP-qPCR) and focused on three loci at *Xist* 5′-regions containing the major promoter and repeat A, which is essential for establishment of iXCI^26^ (Fig. 4b). We first tested whether the quantification system was biased by using diluted DNA from bulk ES cells. The results of ChIP-qPCR from pre-amplified DNA were comparable to those obtained without pre-amplification (Supplementary Fig. 8).

Using this system, we examined H3K9me3 states at three *Xist* regions and at *Gapdh* promoter regions (as a negative control) in PEs at the four-cell stage. We first investigated whether our eChIP method was efficient by examining major satellite repeats that have been identified as H3K9me3-rich regions in preimplantation embryos^23^. Consistent with a previous report, H3K9me3 was highly enriched at major satellite regions (Fig. 4c). The three *Xist* regions were also highly methylated compared with the *Gapdh* promoter region, as follows: 5′-R, 3.7-fold upregulated; XP, 9.8-fold upregulated; RA, 12.1-fold upregulated; (Fig. 4d).

As Fig. 2a showed Xm-*Xist* spontaneous derepression at the morula stage, we next investigated whether H3K9me3 levels at the *Xist* promoter region were low at this stage. eChIP-aPCR analysis revealed that enrichment of H3K9me3 was markedly reduced compared with that at the *Gapdh* promoter region in the four-cell stage (5′-R, 0.83-fold upregulated; XP, 2.85-fold upregulated; RA, 4.8-fold upregulated; Fig. 4e). These results suggested that demethylation at the promoter region was essential for Xm-*Xist* derepression.

We then asked whether *Xist* promoter demethylation was involved in the Xm-*Xist* derepression observed in *Kdm4b*-PEs at the four-cell stage. The H3K9me3 levels at major satellite regions in *Kdm4b*-PEs were significantly reduced compared with those in *Egfp*-PEs (*Kdm4b*-PEs: 9.74% versus *Egfp*-PEs: 24.63%, *P*<0.01, Student’s *t*-tests; Fig. 4f). At three *Xist* regions, the H3K9me3 levels of the promoter region in *Kdm4b*-PEs were significantly reduced, as follows: 5′-R, *Kdm4b*-PEs: 0.84% versus *Egfp*-PEs: 11.48%, *P*<0.05, Student’s *t*-tests; XP, *Kdm4b*-PEs: 3.13% versus *Egfp*-PEs: 16.08%; *P*<0.04, Student’s *t*-tests; and RA, *Kdm4b*-PEs: 20.16% versus *Egfp*-PEs: 29.99%; Fig. 4g. Taken together, these results demonstrated that H3K9me3 at the promoter region protected Xm-*Xist*, preventing RNF12-mediated activation from the four-cell stage. We concluded that silencing of Xm-*Xist* by imprinting to establish iXCI involved H3K9me3.

### Maternal repressive H3K9me3 mark is absent in ES cells

Previous studies have shown that *Xist* is ectopically expressed in embryos cloned from somatic and ES cells^27^,^28^. However, the cause of aberrant *Xist* expression in cloned embryos remains unknown. Given that high H3K9me3 levels at the promoter region in PEs are lost during development (Fig. 4d,e), we investigated whether the maternal repressive H3K9me3 mark was lost in ES cells and whether *Xist* in ES cells was permissive against RNF12 during oocyte-mediated reprogramming.

To test this possibility, we first examined H3K9me3 states at *Xist* regions in various types of male ES cells, using published data^29^,^30^. The levels of H3K9me3 at *Xist* regions containing promoters in TT2 and E14 ES cell lines were low compared with those in positive control regions (Supplementary Fig. 9). ChIP-seq analysis revealed that although ectopic expression of KDM4B in male ES cells induced global H3K9me3 demethylation (Supplementary Fig. 10a–d), it did not alter H3K9me3 levels at *Xist* regions. Moreover, these levels were low compared with those of a known H3K9me3-rich region (Supplementary Fig. 10e)^16^. Furthermore, the expression of *Xist* in cloned embryos was also not affected by ectopic expression of KDM4B (Supplementary Fig. 10f). These results indicated that the maternal repressive H3K9me3 mark was lost.

To establish whether RNF12 is involved in *Xist* activation during oocyte-mediated reprogramming, oocytes treated with si-*Rnf12* were used as recipients for nuclear transfer (Supplementary Fig. 10g). At the four-cell stage, derepression of *Xist* transcription in ES-cloned embryos depended on RNF12 (>50% repression in si-*Rnf12* group; Supplementary Fig. 10h). Taken together, these data indicated that the intrinsic H3K9me3 mark, which was essential for repression of *Xist* by RNF12, was lost during embryo development. This indicated that the primary cause of aberrant *Xist* expression in cloned embryos was involved in loss of intrinsic H3K9me3 at *Xist* regions.

### Effects of iXCI disruption on FEs

The effects of Xm-*Xist* derepression on postimplantation development remain unclear. First, we asked whether ectopic *Kdm4b* expression caused Xm-*Xist* derepression in FEs. *Kdm4b*-FEs developed into blastocysts with high efficiency (>80% of two-cell embryos; Supplementary Table 6). At the 96-h blastocyst stage, *Xist* transcription was derepressed in male *Kdm4b*-FEs, while *Pgk1* and *Plac1* levels were reduced to <13% of those observed in controls (Supplementary Fig. 11a). In female *Kdm4b*-FEs, FISH analysis revealed that there were cells with *Xist* biallelic expression (Supplementary Fig. 11b). Although the expression level was slightly elevated (1.3-fold), X-linked genes were also significantly repressed in female *Kdm4b*-FEs (Supplementary Fig. 11c). These results showed that ectopic Xm-*Xist* derepression caused X-linked gene silencing and elimination of iXCI.

To test the effects of iXCI disruption on postimplantation development, we conducted *in vivo* transplantation experiments. Interestingly, our results demonstrated that XCI on Xm during preimplantation did not affect developmental competence to term (*Kdm4b*-FEs: 63.2% versus *Egfp*-FEs: 53.1%; Supplementary Fig. 11d,e), suggesting that aberrant XCI in preimplantation embryos was restored during postimplantation development, probably through an automatic counting function. These results were consistent with the observation that the developmental competency of embryonic cloned embryos was high, despite the ectopic expression of *Xist* and the occurrence of global XCI (Supplementary Fig. 12)^28^,^31^.

### Loss of XCI impairs the postimplantation development of PEs

It is still unknown whether the embryonic lethality observed immediately after implantation in the majority of PEs (around 70–80%) can be attributed to the loss of dysregulation of X-linked genes or to loss of expression of autosomal paternally imprinted genes. Figure 2 showed that the percentage of *Xist*-positive cells in *Kdm4b*-PEs was significantly higher than that in *Egfp*-PEs. Thus, we reasoned that *Kdm4b*-PEs would be suitable for studying this long-standing question.

**Figure 5:**
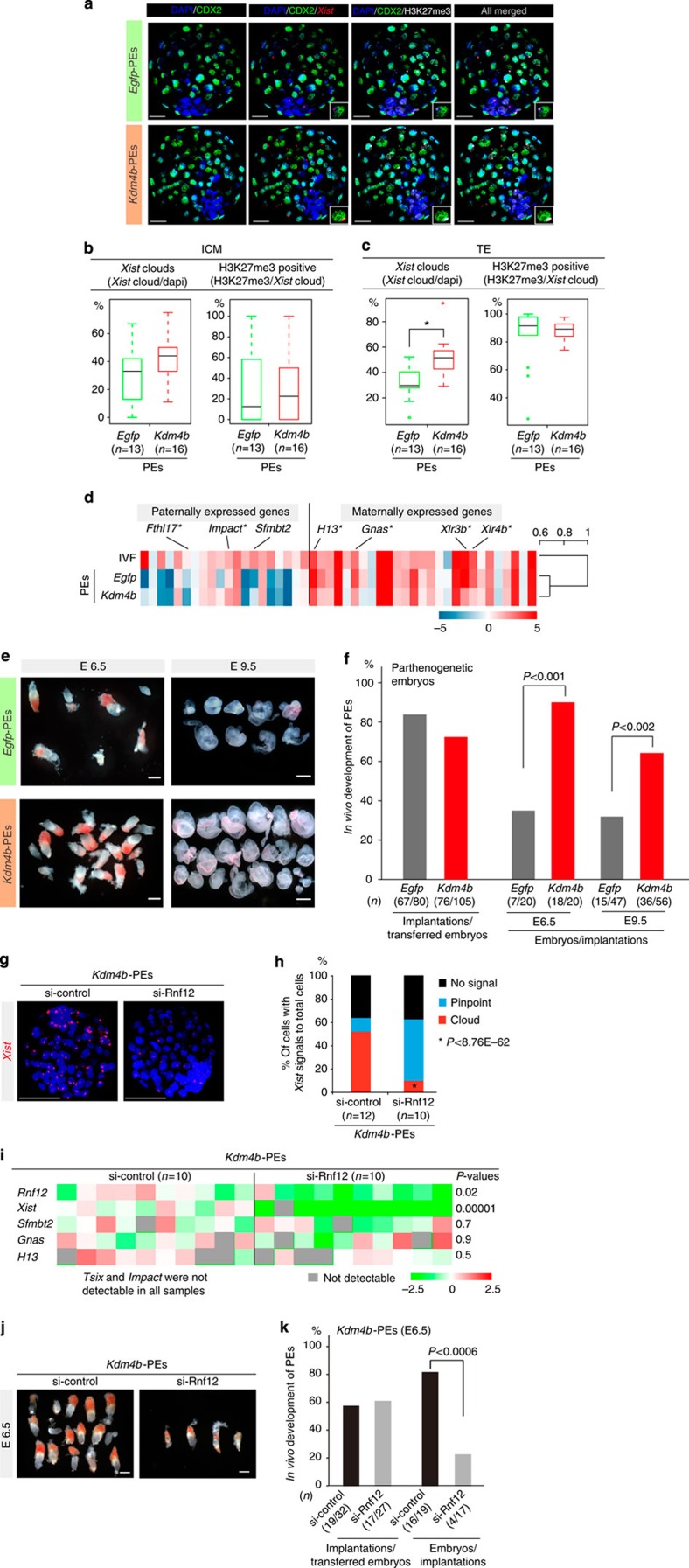
Loss of Xm-XCI is the primary cause of developmental failure immediately after implantation in most PEs. (**a**) IF combined with FISH analysis of blastocysts in *Egfp*-PEs (upper panel) and *Kdm4b*-PEs (lower panel). CDX2-positive cells were identified as belonging to the trophectoderm (TE). Representative pictures of Z-sections. 4′,6-diamidino-2-phenylindole (DAPI) (blue), CDX2 (green), *Xist* (red) and H3K27me3 (white). Scale bars, 20 μm. The rates of cells with *Xist* (left) or H3K27me3 (right) in the inner cell mass (ICM) (**b**) and TE (**c**), respectively. *n*, number of embryos analysed. The number of cells analysed is shown in Supplementary Table 7. *P<4.3 × 10^−23^ (Fisher’s exact test). (**d**) Expression states and clustering analysis of imprinted genes. *Sfmbt2* important for placentation and differentially expressed genes (asterisk) are shown. The scale bar indicates normalized values of log_2_. (**e**) Embryos with extra-embryonic tissues at E6.5 and E9.5 for *Kdm4b*- and *Egfp*-PEs, respectively. Upper and lower images indicate *Egfp*- and *Kdm4b*-PEs, respectively. Left and right column sides show E6.5 and E9.5, respectively. Scale bars, 200 μm (E6.5) and 500 μm (E9.5). (**f**) Summary of the developmental abilities of *Kdm4b*-PEs and *Egfp*-PEs at postimplantation stages (E6.5 and E9.5). Five and 12 independent recipients were analysed at E6.5 and E9.5, respectively. (**g**,**h**) *Xist* analysis in *Rnf12-*knockdown and control *Kdm4b*-PEs. Representative images of FISH analysis. Scale bars, 50 μm (**g**) and *Xist* expression states (**h**). (**i**) Expression of imprinted and X-linked genes in *Rnf12*-knockdown and control *Kdm4b*-PEs. *P*-values were determined using Student’s *t*-tests. (**j**) Embryos with extra-embryonic tissues at E6.5 in *Rnf12*-knockdown and control *Kdm4b*-PEs. Scale bars, 200 μm. (**k**) Summary of the developmental ability of *Rnf12*-knockdown and control *Kdm4b*-PEs at E6.5. Five independent recipients were analysed. The *P*-values were determined using Fisher’s exact test.

We first performed a detailed analysis of Xm-XCI states in *Kdm4b*-PEs using IF against H3K27me3 and CDX2, a marker of the trophectoderm, in combination with *Xist* FISH, at the blastocyst stage. This analysis revealed that *Xist*-positive cells were significantly increased in the trophectoderm of *Kdm4b*-PEs, although the ratio of H3K27me3-positive cells was comparable to that of *Egfp*-PEs (Fig. 5a–c and Supplementary Table 7). However, no significant difference was observed in the inner cell mass between groups (Fig. 5a–c and Supplementary Table 7), indicating that loss of H3K9me3 in the maternal genome led to establishment of Xm-XCI as an imprinted Xp-XCI.

Next, we carried out transcriptome analysis in *Egfp*-PEs, *Kdm4b*-PEs and FEs using microarray. Clustering analysis based on gene expression patterns showed that all three groups could be distinguished clearly from each other (Supplementary Fig. 13a). Comparison of transcripts between *Egfp*-PEs and *Kdm4b*-PEs identified transcripts that were significantly differentially expressed: 671 transcripts were upregulated and 711 transcripts were downregulated (*P*<0.05, Student’s *t*-test and>1.5-fold changes in *Kdm4b*-PEs). Chromosome distribution analysis showed that upregulated transcripts in *Kdm4b*-PEs were distributed across various chromosomes (2.33–7.23%; Supplementary Fig. 13b). However, downregulated transcripts in *Kdm4b*-PEs were mostly concentrated on the X chromosome, which particularly involved the *Xlr* and *Magea* families (10.26%; Supplementary Fig. 13c–e).

Comparison of the imprinted genes between *Egfp*- and *Kdm4b*-PEs revealed that only six genes were significantly differentially expressed (paternally expressed genes: *Impact* and *Fthl17*; maternally expressed genes: *Gnas*, *H13*, *Xlr3b* and *Xlr4b*; Fig. 5d). Clustering analysis based on the expression of imprinted genes showed that the expression levels in *Kdm4b*-PEs were similar to those in *Egfp*-PEs rather than to those of biparental embryos (Fig. 5d). Thus, H3K9me3 demethylation does not result in restoration of expression states in paternally expressed genes.

We conducted *in vivo* transplantation experiments using *Kdm4b*-PEs. Surprisingly, at E6.5, the developmental rates of *Kdm4b*-PEs were markedly increased compared with those of *Egfp*-PEs (*Kdm4b*-PEs: 90% versus *Egfp*-PEs: 35%; *P*<0.001, Fisher’s exact test; Fig. 5e,f). At E9.5, although the stages of the recovered embryos varied, *Kdm4b*-PEs retained a significantly higher developmental ability compared with controls (*Kdm4b*-PEs: 64.3% versus *Egfp*-PEs: 31.9%; *P*<0.002, Fisher’s exact test; Fig. 5e,f).

However, we did not rule out the possibility that the significant improvement in *Kdm4b*-PE development resulted from restoration of the expression levels of some imprinted genes. To determine whether the improvement in developmental competency could be attributed to the gain of XCI, we constructed *Kdm4b*+si-*Rnf12*-PEs. In *Kdm4b*-PEs with si-*Rnf12* at the blastocyst stage, *Xist* expression analysis by FISH revealed that *Xist* cloud signals in control *Kdm4b*-PEs were present in 51.9% of cells, while those in *Rnf12*-knockdown *Kdm4b*-PEs were present in only 10% of cells, and most of the signals were pinpoint rather than cloud (Fig. 5g,h and Supplementary Table 8).

qPCR analysis showed that although *Xist* signals were significantly reduced in *Kdm4b*-PEs with si-*Rnf12* (12.5% of the control on average), the expression levels of *Impact*, *H13* and *Gnas*, which are expressed in response to ectopic *Kdm4b* expression (Fig. 5d), did not change when compared with those of controls (Fig. 5i). We further demonstrated that RNF12 depletion did not affect *Tsix* and *Sfmbt2* expression levels in *Kdm4b*-PEs (Fig. 5i). These results clearly indicated that RNF12 depletion led to *Xist* downregulation in *Kdm4b*-PEs, without altering the features of PEs.

Finally, *in vivo* transplantation experiments demonstrated that *Xist* repression by RNF12 depletion significantly inhibited developmental competency at E6.5 in *Kdm4b*-PEs (*Kdm4b*+si-control: 84.2% versus *Kdm4b*+si-*Rnf12*: 23.5%; *P*<0.0006, Fisher’s exact test; Fig. 5j,k). Taken together, these results demonstrated that the developmental defects seen in PEs immediately after implantation could be attributed to the lack of XCI, but not to loss of expression of paternally expressed genes.

## Discussion

In this study, we demonstrated that maternal imprinting of Xm, which protected against *Xist* activation by RNF12 in the preimplantation stages, was mediated by H3K9me3.

Xm-*Xist* imprints are established during oogenesis and autosomal imprinting also occur in the phases^6^,^10^. In many imprinted genes, DNA methylation at the promoter regions is the primary regulator and H3K9me3 modifications overlap with these regions^32^. However, it is not clear why Xm-*Xist* regions are targeted by H3K9me3, but not by DNA methylation. One of the possibilities is that during primordial germ cell development, *Xist* must be silenced to activate the inactivated allele before inducing the expression of *Dnmt3a*/*3l*, which encodes a *de novo* DNA methyltransferase that is activated during oogenesis^33^. Consistent with this concept, *Xist* repression begins in primordial germ cells at E10.5 (ref. 34). Thus, comparison of H3K9me3 states at promoter regions in non-growing oocytes with those in growing oocytes will greatly facilitate understanding of the molecular mechanisms of iXCI.

We found that *Kdm4b*-, *Egfp*+TSA- and *Kdm4b*+TSA-PEs did not show complete XCI at the blastocyst stage as compared with female FEs. These results suggested that other repressive marks were imposed on Xm to silence Xm-*Xist* expression. Alternatively, removal of H3K9me3 may be incomplete because demethylation at RA regions was mild (Fig. 4g). However, it is not clear why RA regions show resistance against demethylation by KDM4B. As suggested in a previous study, this mechanism may be related to the three-dimensional structure of the A-repeat, which has been reported to constitute stable regions in the *Xist* transcript^35^. Further studies using ChIP-seq and/or chromatin-conformation capture sequencing technologies in preimplantation embryos are required for comprehensive understanding of *Xist* regulation.

In ES cells, RNF12 induces *Xist* expression through degradation of REX1, which is required for suppression of *Tsix*^36^. Interestingly, we did not detect *Tsix* expression from the morula to the blastocyst stages in *in vitro*-fertilized (IVF) embryos, implying that the molecular mechanism of RNF12-mediated *Xist* activation differs between imprinted and randomly induced XCI. It is not known whether the role of RNF12 in *Xist* activation during the preimplantation stages was direct or indirect. Recent RNF12 studies reported the specific binding of RNF12 to *Smad7* in mouse ES cells^37^, suggesting that signalling via transforming growth factor-β family members may be associated with imprinted *Xist* activation.

In this study, we revealed the molecular mechanisms underlying imprinting of XCI and demonstrated the role of XCI in various types of embryo development in mice. Recent studies using somatic- and ES-cloned embryos revealed that aberrant *Xist* reprogramming is a major cause of developmental failure in cloned embryos^27^,^28^. We found that RNF12 was highly expressed in oocytes compared with somatic and ES cells (>80-fold). Moreover, we showed that H3K9me3 levels at *Xist* promoter regions were low in ES cells and that *Xist* expression in ES-cloned embryos depended on RNF12. These data provided the first evidence that RNF12 inhibited developmental reprogramming. Therefore, the use of RNF12-depleted oocytes as recipient cells would improve cloning efficiency. However, *Xist* activation in cloned embryos may be induced by factors other than RNF12, as supported by the observation that *Xist* was still expressed at ~40% of control levels, even after marked depletion of RNF12. Consistent with this notion, a recent study has demonstrated that RNF12 is dispensable for random XCI *in vivo*^38^.

Xm-*Xist* derepression from the four-cell stage could rescue developmental defects in PEs. This finding demonstrated that the primary cause of developmental failure immediately after implantation was a lack of XCI, but not a lack of expression of paternally imprinted genes. We also tested whether *Kdm4b*-PEs could extend development; however, we did not observe extended *Kdm4b*-PE development after E9.5, implying that expression of paternally imprinted genes is required for subsequent development in PEs^11^,^12^.

Our data resolved several long-standing unanswered questions about XCI during preimplantation in various types of embryos (Supplementary Fig. 14). Moreover, given that injection of *Kdm4b* mRNA into PEs improved their developmental ability, genetic mutation leading to embryonic lethality could be rescued by transient expression of epigenomic modifiers during preimplantation phases.

## Methods

### Embryo manipulations

All mice were maintained and used in accordance with the Guidelines for the Care and Use of Laboratory Animals of the Japanese Association for Laboratory Animal Science and the National Research Institute for Child Health and Development (NRICHD) of Japan. All animal experiments were performed according to protocols approved by the Institutional Animal Care and Use Committee of the NRICHD (Permit Number: A2006-009).

Adult female B6D2F1 mice were purchased from Clea Japan (Tokyo, Japan) and oocytes were collected following standard methods^27^. PEs were generated using Ca-free M16 medium containing 8 mM SrCl_2_ and Cytochalasin B (5 μg ml^−1^) (Sigma-Aldrich, St Louis, MO, USA), and cultured KSOM (EMD Millipore, Darmstadt, Germany). Injection experiments (mRNA, short interfering RNA (siRNA) and nuclear transfer) were conducted using a Prime Tech Piezo drive (Sutter Instrument Company, Novato, CA, USA). To produce cloned embryos, nuclear-transferred oocytes were parthenogentically activated. Manipulated embryos were cultured to the developmental stages, as follows: 4-cell, 48 h; morula, 72 h; and blastocyst, 96 and 120 h after parthenogenetic activation or ICSI, respectively. All embryos were cultured at 37 °C in KSOM in an atmosphere containing 5% CO_2_. In the TSA experiment, the embryos were cultured for 24 h in activation and culture media containing 50 nM TSA (Sigma-Aldrich). IVF fertilization and nuclear transfer were performed following published procedures^27^. To determine the effects of ectopic KDM4B expression on *Xist* expression in cloned embryos, doxycycline was added to ES cell culture and KSOM medium to a final concentration of 2 μg ml^−1^. Pseudopregnant ICR mice (Clea Japan) were used as embryo recipients. At E6.5, E9.5 and E18.5, the embryos were recovered from the uterus.

### *In vitro* mRNA synthesis

The coding region of *Kdm3a* was amplified from mouse testis complementary DNA using PCR with KOD-Plus-Neo DNA polymerase (Toyobo, Osaka, Japan). Forward and reverse primers contained T7 promoter and poly(T)_120_ sequences, respectively. A step-down PCR amplification method was used, following the manufacturer’s instructions (Toyobo). Poly(A)-containing PCR products were subjected to *in vitro* transcription using a mMESSAGE mMACHINE T7 ULTRA Kit (Life Technologies, Carlsbad, CA, USA), following the manufacturer’s instructions. To generate a *Kdm4b* DNA template for *in vitro* transcription, pCMV-SPORT6 containing the full-length *Kdm4b* mRNA was used as the PCR template (DNAFORM, Kanagawa, Japan, Clone ID 3490671). *Egfp* cDNA was cloned using the pGEM-T Easy Vector System (Promega, Madison, WI, USA) and transcribed *in vitro* using the mMESSAGE mMACHINE T7 ULTRA Kit (Life Technologies) following the manufacturer’s instructions. The concentrations of the mRNAs were adjusted to 150 ng ml^−1^ (*Egfp*), 550 ng ml^−1^ (*Kdm3a*), or 450 ng ml^−1^ (*Kdm4b*) to maintain a constant number of injected mRNA molecules. The primer sequences used for generating the templates for *in vitro* transcription are shown in Supplementary Table 9.

### *Rnf12* knockdown

siRNA targeting *Rnf12* (si-Rnf12 sense 5′-GAAGUCAAAUGGAUCGCUUTT-3′ A and antisense 5′-AAAGCGAUCCAUUUGACUUCTG-3′ GC, and the negative control siRNA (si-control: 4390846) were purchased from Life Technologies. The final concentration of each siRNA was 50 ng ml^−1^. The siRNA was injected into MII oocytes using the Piezo drive and then incubated for 6–7 h in KSOM medium at 37 °C in an atmosphere containing 5% CO_2_ before mRNA injection. For the NT experiment using *Rnf12*-knockdown oocytes, oocytes were incubated for 5–6 h after siRNA injection, and NT was then conducted and activated as described above.

### Immunofluorescence

Oocytes injected with mRNAs were subjected to ICSI. After 10–11 h, the zygotes were fixed with 2% paraformaldehyde (PFA) in PBS containing 0.1% polyvinyl alcohol (PBS-PVA) for 15 min at room temperature (RT). Zygotes were then permeabilized using 0.2% Triton X-100 in PBS-PVA for 15 min at RT and blocked in 1% BSA in PBS-PVA for 1 h at RT. The primary antibodies used in the assay were as follows: anti-H3K9me3 (ab8898, 1:500 dilution, Abcam, Cambridge, UK), anti-H3K9me2 (ab1220, 1:500, Abcam) and anti-H3K27me3 (07-449, 1:150, EMD Millipore). The primary antibodies were diluted with blocking solution (PBS-PVA containing 1% BSA) and the embryos were incubated overnight at 4 °C. After washing in blocking solution, the embryos were incubated for 1 h at RT with Alexa Flour 634- or 546-conjugated anti-mouse or anti-rabbit IgG secondary antibodies (1:500, Life Technologies). After the embryos were washed, the nuclei were stained with 1 μg ml^−1^ 4′,6-diamidino-2-phenylindole and the embryos were placed on a glass slide and observed with a LSM510 laser scanning confocal microscope (Carl Zeiss, Oberkochen, Germany). Signal intensities of maternal and paternal pronuclei were calculated using NIH ImageJ software ( http://rsb.info.nih.gov/ij/).

In *Rnf12-*knockdown experiments, one-cell and four-cell PEs were fixed at 10–11 h (18–19 h after siRNA injection) and 48 h after activation, respectively. Anti-RNF12 (1:500, Abnova, Taipei, Taiwan) and Alexa Fluor 488-conjugated anti-mouse IgG antibodies (1:500, Life Technologies) were used as the primary and secondary antibodies, respectively. *Rnf12-*knockdown and negative-control PEs were observed under the same conditions, to assess knockdown efficiency. Signal intensities were calculated using ImageJ software.

### Fluorescent *in situ* hybridization

The zona pellucida of embryos was removed using acid Tyrode solution (Sigma-Aldrich) and then fixed and permeabilized with 2% PFA-PVA containing 0.25% Triton X-100 for 10 min on ice. The samples were placed on glass slides, evaporated to dryness, dehydrated sequentially in 70 and 100% ethanol and then air-dried. Hybridization buffer containing an *Xis*t probe (provided by T. Sado) was prepared using a Nick Translation Kit (Abbott, Abbott Park, IL, USA) and Cy3-dUTP (GE Healthcare Life Sciences, Fairfield, CT, USA) and was then applied to the slides. The slides were then incubated and washed as previously described^26^. Fluorescence was visualized using the LSM510.

### IF combined with FISH

The zona pellucida of embryos was removed using acid Tyrode solution (Sigma-Aldrich) and fixed with 2% PFA-PVA for 15 min at RT in four-well dishes. The fixed samples were permeabilized with 0.5% Triton X-100 in PBS-PVA for 20 min on ice. After washing with PBS-PVA, the samples were blocked in 1% BSA-PBS-PVA containing 1.3 U ml^−1^ RNaseOUT (Life Technologies) for 30 min at RT. After washing, the embryos were incubated with primary antibodies (anti-CDX2 (BioGenex, San Ramon, CA, USA), diluted 1:30 and anti-H3K27me3 diluted 1:150 in blocking buffer containing 1.3 U ml^−1^ RNaseOUT) for 1 h at RT. Secondary antibody reactions were performed as described above. The samples were placed on glass slides, evaporated to dryness, dehydrated sequentially in 70 and 100% ethanol and air dried. The samples were then analysed by FISH according to the procedures described above.

### Analysis of IF combined with FISH data

*Xist* cloud signals detected in three-dimensional images using *Z*-sections of the LSM Image Browser (Carl Zeiss) were judged as positive. Only cells that did not overlap at interphase were used in the analysis. Biallelic expression was defined as cells with two *Xist* cloud spots. Statistical analysis was performed using Fisher’s exact test.

### Gene expression analysis

Total RNA was extracted using an RNeasy Micro Kit (Qiagen, Venlo, The Netherlands) and treated with DNase following the manufacturer’s instructions. mRNAs were reverse transcribed using an oligo(dT) primer and SuperScriptIII Reverse Transcriptase (Life Technologies). For quantitative gene expression analysis with high specificity, TaqMan probes (Life Technologies) were used in all assays. In four-cell stage embryos, *Xist* was assayed in triplicate and only the samples that were detected in two or three replicates were judged as positive. In morulae and blastocysts, expression of target genes was assayed in duplicate. *Gapdh* was used as the internal control in the four-cell-stage assays and *Rnf12* was used in the time-lapse assays. *Gapdh* and *Actb* (encoding β-actin) were used as internal controls at the morula and blastocyst stages. For normalization of qPCR analysis (Fig. 2b,g), the expression levels of all embryos were normalized to the average expression levels of *Egfp*-PEs. The TaqMan probes and primer sets used in this study are shown in Supplementary Table 8.

### Generation of *Kdm4b*-inducible ES cell lines and ES cell culture

The XhoI- and ClaI-linearized pGEM-IRES-EGFP plasmids were inserted into the cognate sites of pPB-CAG-EBNX (provided by A. Bradley) to generate pPB-CAG-IRES-EGFP. A Tet3G fragment with BglII and XhoI cleavage sites was amplified from a pEF1a-Tet3G template (Clontech Laboratories, Mountain View, CA, USA) using PCR and inserted into pPB-CAG-IRES-EGFP, generating the vector pPB-CAG-Tet3G-IRES-EGFP. The XhoI and BamHI cleavage sites in pPB-UbC (provided by A. Bradley) were replaced with the p-TRE3G multiple cloning sites (Clontech). The *Kdm4b* coding sequence, with terminal ClaI and BamHI cleavage sites, was amplified by PCR and inserted into the corresponding sites of pPB-TRE3G, yielding pPB-TRE-Kdm4b.

The NCH.4.6 male mouse ES cell line (C57B6/N × C57B6/N), which had a normal karyotype, was electroporated with pPB-TRE-Kdm4b, pPB-CAG-Tet3G-IRES-EGFP and pCMV-hyPBase (provided by A. Bradley). All ES cells used in this study were cultured in knockout DMEM (Life Technologies) containing recombinant human leukemia inhibitory factor culture supernatant for mouse ES cell culture (Wako Pure Chemical Industries, Ltd, Osaka, Japan), as well as GlutaMAX, 2-mercaptoethanol, non-essential amino acids and 15% KSR (all from Life Technologies). Doxycycline (2 μg ml^−1^; Sigma-Aldrich) was added to ES cell culture medium to induce ectopic KDM4B expression.

### Western blotting

Cells were extracted using a stock lysis buffer containing 1 M Tris–HCl, 5 M NaCl, 10% Triton-X and protease inhibitors, and were subjected to e-PAGEL (ATTO, Amherst, NY, USA) electrophoresis. The membranes were washed in TBS containing 0.1% Tween 20 (TBS-T) and blocked in 5% skim milk in TBS-T for 1 h. The membranes were incubated with anti-KDM4B antibodies (1:500 dilution; Active Motif, Carlsbad, CA, USA) overnight at 4 °C, washed and incubated with a rabbit horseradish peroxidase-conjugated secondary antibody (1:5,000 dilution; Sigma-Aldrich) for 1 h at RT. Immunoblottings were visualized using SuperSignal chemiluminescent substrate (Thermo Scientific, Waltham, MA, USA) and an ImageQuant LAS4000 system (GE Healthcare). After capturing the images, the membranes were washed with WB Stripping Buffer (Nacalai Tesque, Kyoto, Japan) for 10 min, washed with TBS-T and incubated with an anti-β-actin antibody conjugated to fluorescein isothiocyanate (1:2,000 dilution; Sigma-Aldrich) for 1 h at RT.

### ChIP analysis of K4B-ES cells

Trypsinized feeder-free ES cells (2 × 10^7^) were collected and fixed with 1% formaldehyde. The cells were resuspended in SDS lysis buffer (ChIP Reagent, Nippon Gene Co., Ltd.) and the lysate was sonicated to fragment chromatin using a S220 Focused-ultrasonicator (Covaris, Woburn, MA, USA). The chromatin was purified by centrifugation and immunoprecipitated with Protein A-beads (Veritas Life Sciences, Ribeirão Preto, Brazil) conjugated to anti-H3K9me3 antibodies (Abcam: ab8898) or rabbit IgG (Abcam: ab37415) in Buffer A with protease inhibitor (LowCell ChIP kit, Diagenode, Denville, NJ, USA) overnight at 4 °C. The chromatin beads were washed with Buffers A and C (LowCell ChIP kit). After washing, the chromatin beads were incubated in ChIP direct elution buffer (ChIP Reagent) for 6 h at 65 °C, following incubation with 2 μl proteinase K (20 mg0 ml^−1^) for 2 h at 55 °C. The DNA immunoprecipitated from the supernatant was purified using Agencourt AMPure XP beads (Beckman Coulter, Inc., Pasadena, CA, USA) according to the manufacturer’s instructions.

### ChIP combined with deep sequencing

ChIP-Seq libraries were prepared using the NEBNext ChIP-Seq Library Prep Master Mix Set and Multiplex Oligos from Illumina (New England BioLabs Inc., Ipswich, MA, USA) according to the manufacturer’s instructions. Ten nanograms of ChIP or input DNA was subjected to end repair, dA-tailing and adaptor ligation, and amplified using nine cycles of PCR. The final library size was checked using a 2100 Bioanalyzer (Agilent Technologies, Santa Clara, CA, USA). After the concentration of each library was determined using qPCR with a KAPA Library Quantification Kit-Illumina/Universal system (KK4824, Kapa Biosystems, Wilmington, MA, USA), the libraries were sequenced using the HiSeq 1000 sequencing system (Illumina, San Diego, CA, USA) to generate 100 bp × 2 paired-end data.

### ChIP-seq data analysis

Reads from each sample were first trimmed by removing adapter sequences and low-quality bases at ends using Trimmomatic 0.22 ( http://www.usadellab.org/cms/index.php?page=trimmomatic). Approximately 115 million reads for each of the ChIP and input libraries were aligned to the mouse reference genome (mm10: http://genome.ucsc.edu/cgi-bin/hgGateway) using the Burrows-Wheeler Aligner 0.6.2. Uniquely mapped reads were selected using a custom script, converted from SAM to BAM format using SAMtools 0.1.18 and processed using Picard 1.83 to mark PCR duplicates. Reads with a mapping quality of <20 were removed using SAMtools 0.1.18. The resulting BAM files (a pair of files for ChIP and input libraries) were visualized using the Integrative Genomics Viewer ( http://www.broadinstitute.org/igv/) and subjected to peak detection using the MACS algorithm implemented in Avadis NGS software (Agilent). In scatter plot analysis using 1 and 15 K4B-ES cell lines, the numbers of mapped reads were counted for 10,000-bp windows (with a sliding size of 5,000 bp). To adjust for differences in total amount of reads, the number of mapped reads in each window was transformed into reads per million format. Calculation methods are available on request.

### ChIP-qPCR analysis of sperm

Sperm were obtained from BDF1 mice aged 9–12 weeks. Preparation of sperm chromatin was performed according to published protocols with modifications^39^,^40^. For each native ChIP experiment, 5 × 10^7^ sperm were used. Sperm were washed twice with PBS. The pellet was suspended in PBS containing 0.5% Triton-X, 10 mM dithiothreitol (DTT) and protease inhibitor (Diagenode), and incubated on ice for 1.5 h. After washing with PBS, pelleted sperm nuclei were suspended in 400 μl PBS containing 1 mM CaCl_2_ and 1 mM DTT, and incubated for 5 min at 37 °C. After incubation, 1 μl (2 × 10^6^ gel units per ml) micrococcal nuclease (New England BioLabs) was added to the nuclei, which were then incubated for 5 min at 37 °C. EDTA was added to a concentration of 0.5 mM and solubilized chromatin was clarified by centrifugation for 15 min at 15,000 r.p.m. at 4 °C. The pellets were suspended in PBS containing CaCl_2_ and DTT (at the same concentrations as used above), and treated again with micrococcal nuclease. To examine whether H3K9me3-modified nucleosomes were present in sperm chromatin, soluble (chromatin) and insoluble (pellet) fractions were subjected to western blotting using anti-H3K9me3 antibodies (ab8898; 1:1,000), as described above.

Chromatin was incubated with Protein A beads conjugated to anti-H3K9me3 antibodies (ab8898) or rabbit IgG (ab37415) overnight at 4 °C in ChIP buffer (40 mM Tris–HCl, pH 7.5, 1 M NaCl and 10 mM EDTA). Pelleted beads were washed twice with Buffer 1 (50 mM Tris–HCl, pH 7.5, 500 mM NaCl and 10 mM EDTA) and Buffer 2 (50 mM Tris-HCL, pH 7.5, 300 mM NaCl and 10 mM EDTA). The pelleted beads were suspended in ChIP direct elution buffer and incubated with proteinase K for 2 h at 37 °C. The immunoprecipitated DNA was then purified using Agencourt AMpure XP beads.

ChIP-qPCR analysis was performed according to published methods using SYBR Green^39^. The sequences of each primer set are listed in Supplementary Table 9.

### eChIP-quantitative qPCR

The zona pellucidae of the embryos were removed by acid Tyrode’s solution and washed in PBS containing 0.1% PVA. The embryos were suspended in PBS containing 0.5% Triton-X, 0.5 mM DTT and protease inhibitor, and incubated on ice for 30 min. After incubation, 1 mM CaCl_2_ was added to the buffer and samples were incubated for 5 min at 37 °C. After incubation, 0.5 μl (2 × 10^6^ gel units per ml) micrococcal nuclease (New England BioLabs) was added to the nuclei, which were then incubated for 5 min at 37 °C. EDTA was added to a concentration of 0.5 mM and solubilized chromatin was clarified by centrifugation at 15,000 r.p.m. for 15 min at 4 °C. The same procedures were repeated one more time. Chromatin was incubated with Protein A beads conjugated to anti-H3K9me3 antibodies (ab8898) or rabbit IgG (ab37415), prepared as described above, overnight at 4 °C in ChIP buffer (40 mM Tris–HCL, pH 7.5, 1 M NaCl and 10 mM EDTA). Pelleted beads were washed twice with Buffer 1 (50 mM Tris–HCL, pH 7.5, 500 mM NaCl and 10 mM EDTA) and then with Buffer 2 (50 mM Tris–HCL, pH 7.5, 300 mM NaCl and 10 mM EDTA). The pelleted beads were then suspended in ChIP direct elution buffer and incubated with proteinase K for 2 h at 55 °C. The immunoprecipitated DNA was then purified using Agencourt AMpure XP beads.

Eluted DNA (20 μl) was divided into two aliquots; one (4 μl) was used for a SYBR Green assay targeting a major satellite and the other (16 μl) was subjected to pre-amplification using a Single Cell-to-CT kit (Ambion, Austin, TX, USA) according to the manufacturer’s instructions. The number of PCR cycles at the pre-amplification step was 20. The primer and probe sequences used are shown in Supplementary Table 9.

### Microarray analysis

Five *Egfp*-PE, *Kdm4b*-PE and IVF blastocysts (120 h) were lysed using ISOGEN (Nippongene) and RNA was extracted by phenol–chloroform and isopropanol precipitation. cDNA was synthesized using the Ovation RNA Amplification System V2 kit (NuGEN, West Cumbria, UK) and hybridized with SurePrint G3 Mouse GE 8x60K Microarray (Agilent Technologies). Analysis was conducted using GeneSpringV12.5 (Agilent Technologies). Transcripts were considered to be expressed if raw values were >100 and a flag was present in at least one of the groups.

## Author contributions

A.F., K.E. and H.A. conceived and designed the study. A.F. performed the experiments and analysis of embryo manipulation, IF, FISH, qPCR, ChIP, cultured ES cells, vector construction and microarray analysis, and developed the eChIP-qPCR technique. J.T., K.H. and K.N. conducted ChIP-seq experiments and analyses. T.M. and H.A. constructed vectors and cultured ES cells. H.A. and A.U. supervised the study. A.F., K.N. and H.A. wrote the manuscript.

## Additional information

**How to cite this article**: Fukuda, A. *et al.* The role of maternal-specific H3K9me3 modification in establishing imprinted X-chromosome inactivation and embryogenesis in mice. *Nat. Commun.* 5:5464 doi: 10.1038/ncomms6464 (2014).

**Accession codes:** The original data for the microarray have been deposited in the GEO at http://www.ncbi.nlm.nih.gov/geo/ (accession number: GSE53662). The original data of ChIP-seq have been deposited in DDBJ at http://cibex.nig.ac.jp/index.jsp with accession number: DRA001041.

## Supplementary Material

Supplementary Figures and Tables Supplementary Figures 1-14 and Supplementary Tables 1-9
