# Supplementary material for: The role of maternal-specific H3K9me3 modification in establishing imprinted X-chromosome inactivation and embryogenesis in mice
Source: Nat Commun. 2014 Nov 14;5:5464. doi: 10.1038/ncomms6464 (PMC4243243; doi:10.1038/ncomms6464)
Supplement: Supplementary Figures and Tables — Supplementary Figures 1-14 and Supplementary Tables 1-9 [file ncomms6464-s1.pdf]

Supplementary Fig. 1

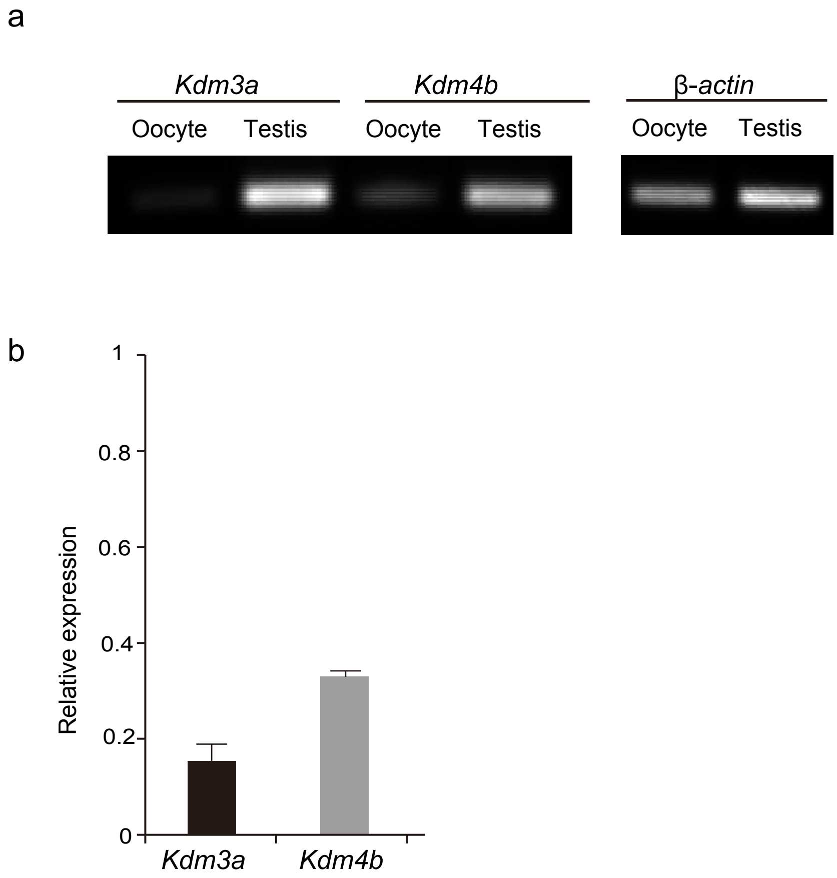

RT-PCR analysis of *Kdm3a* and *Kdm4b* expression in oocytes and testes. Twenty oocytes were pooled for RT-PCR analysis. The number of PCR cycles was 35. Two independent experiments were conducted. (**a**) Representative images of electrophoresis. (**b**) Relative expression levels of *Kdm3a* and *Kdm4b* in oocytes compared with those in testes.

Supplementary Fig. 2

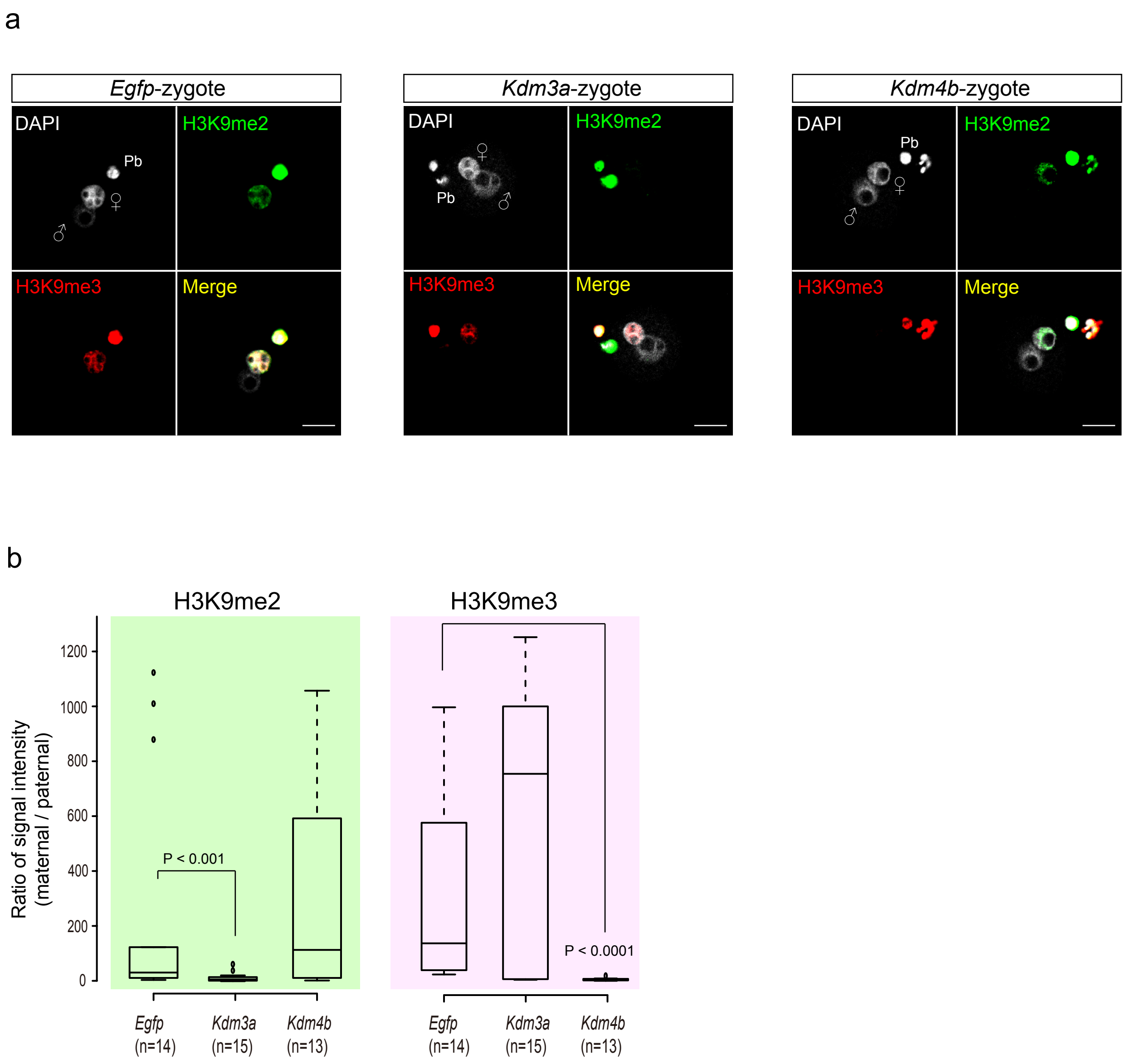

Effects of *Kdm3a* or *Kdm4b* expression on H3K9me2/3 states. (**a**) Representative images of immunofluorescence (IF) analysis in *Kdm3a*- or *Kdm4b*-overexpressing fertilised embryos. (**b**) The box-and-whisker plot shows the ratio of maternal to paternal signal intensities. The *P*-values were calculated using the Mann-Whitney *U*-test (*U*-test) Pb: polar body; *n*, number of embryos analysed. Scale bars = 20 µm.

Supplementary Fig. 3

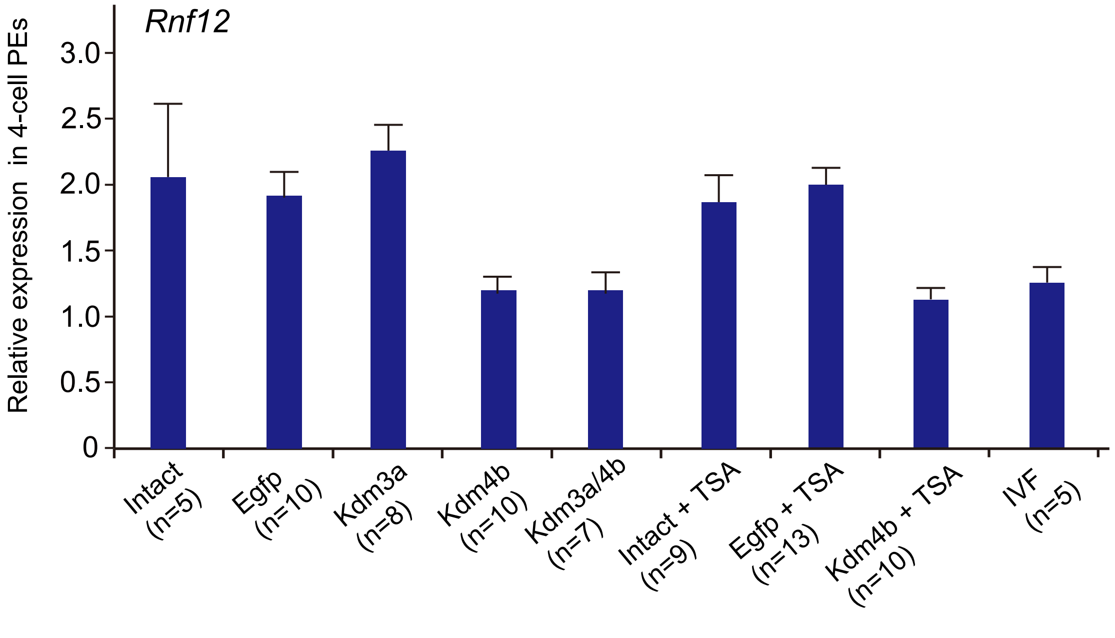

Analysis of *Rnf12* expression using qPCR in parthenogenetic embryos (PEs) harbouring histone modifications and in vitro-fertilised (IVF) embryos at the 4-cell stage. There were no significant differences between groups compared with intact PEs. Error bars indicate the mean ± SEM.

Supplementary Fig. 4

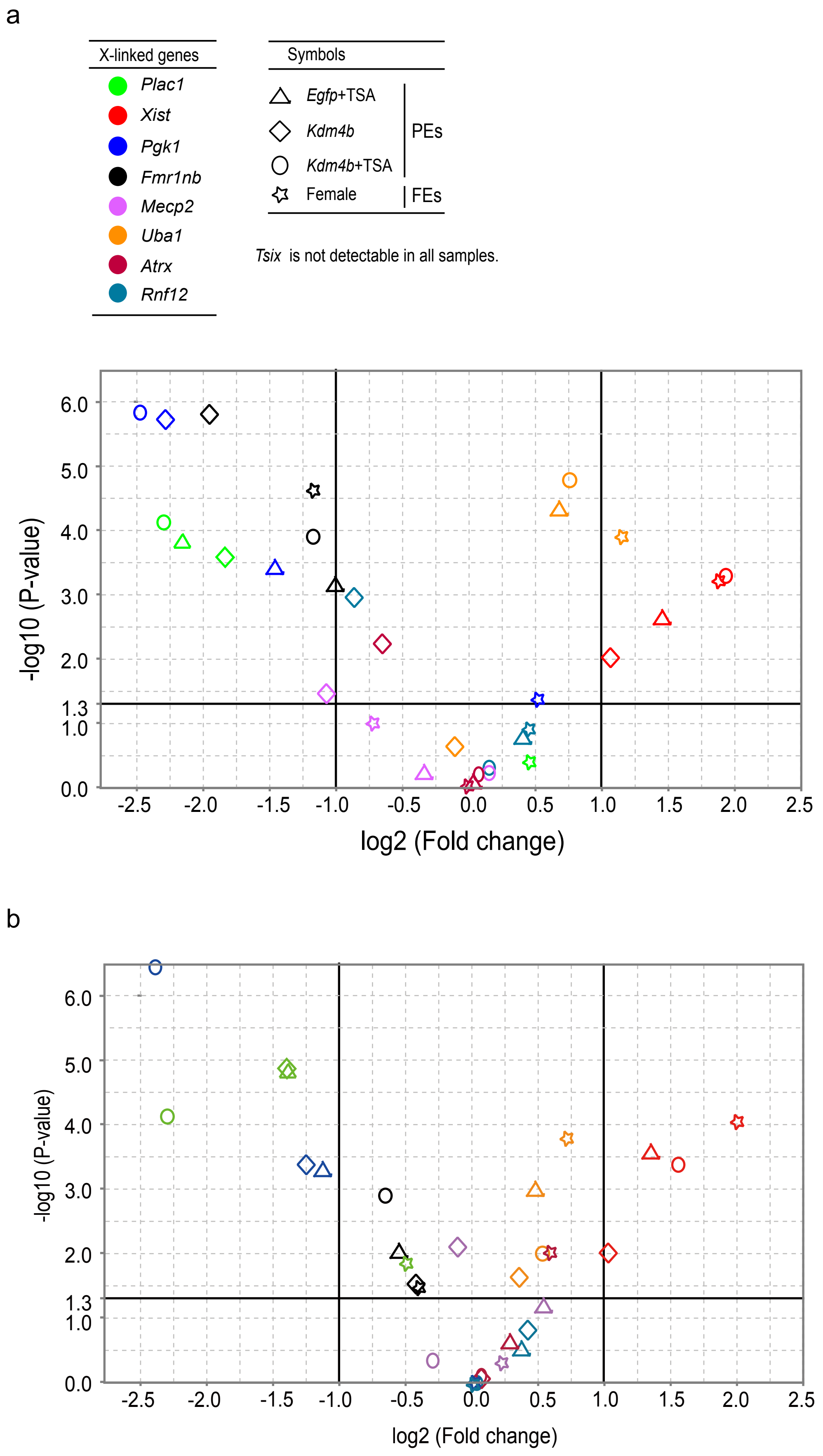

Volcano plot analysis of X–linked genes based on the average expression of each group in 96h and 120h blastocysts. Values are normalised to the average value of *Egfp*-PEs. Vertical and horizontal axes indicate *P*-values and changes in the levels of expression, respectively.

Supplementary Fig. 5

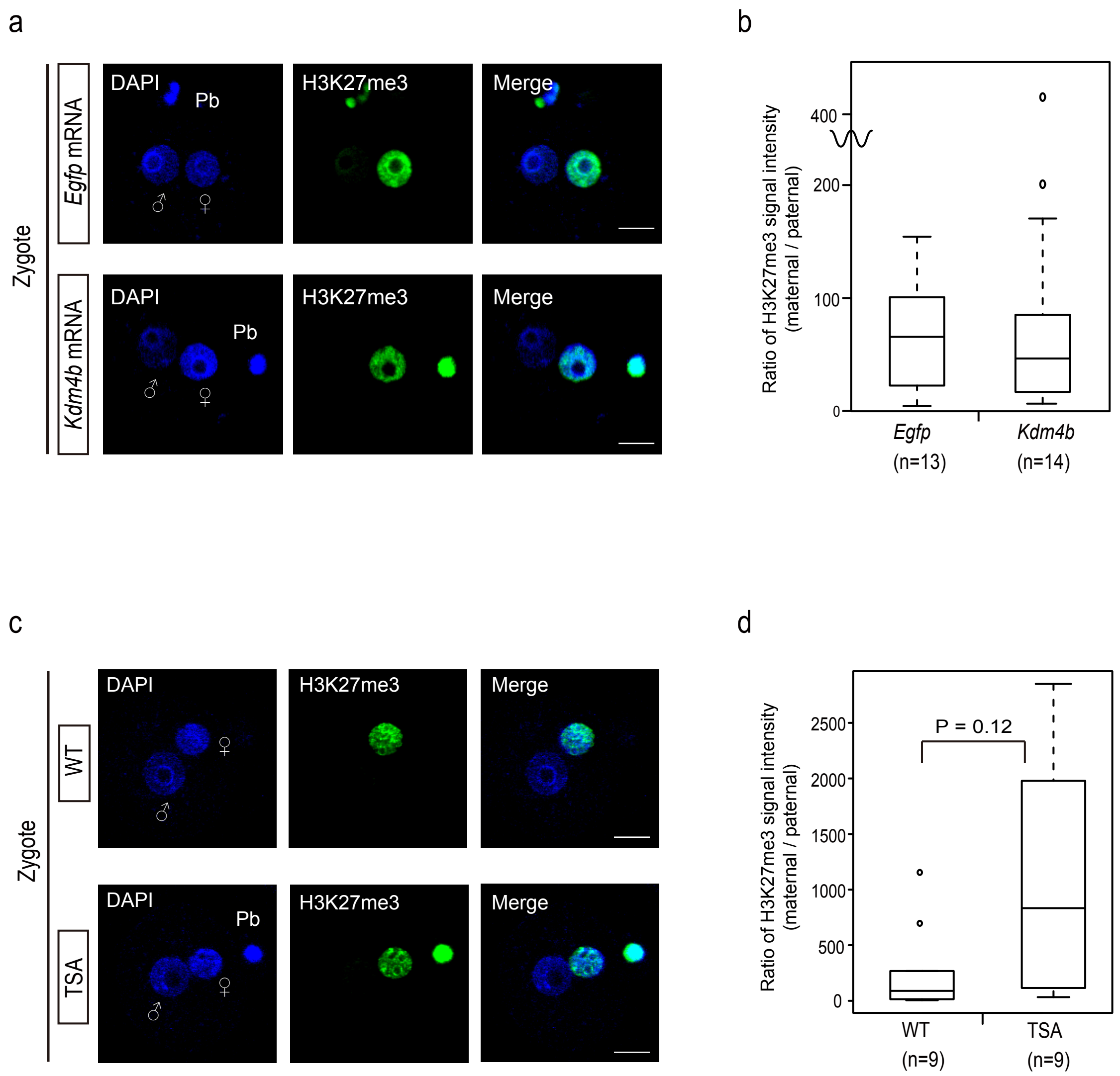

Effects of H3K27me3 modifications on *Kdm4b* overexpression and TSA treatment. (**a**) Oocytes injected with *Kdm4b* mRNA were subjected to ICSI. Samples were fixed 10–11 h after ICSI. (**b**) After ICSI, oocytes were incubated in KSOM along with TSA for 10–11 h, after which the zygotes were fixed. Representative images are shown on the left. The box-and-whisker plot on the right shows the ratio of maternal to paternal signal intensities of H3K27me3. There were no statistically significant differences between groups. Scale bars = 20 μm.

Supplementary Fig. 6

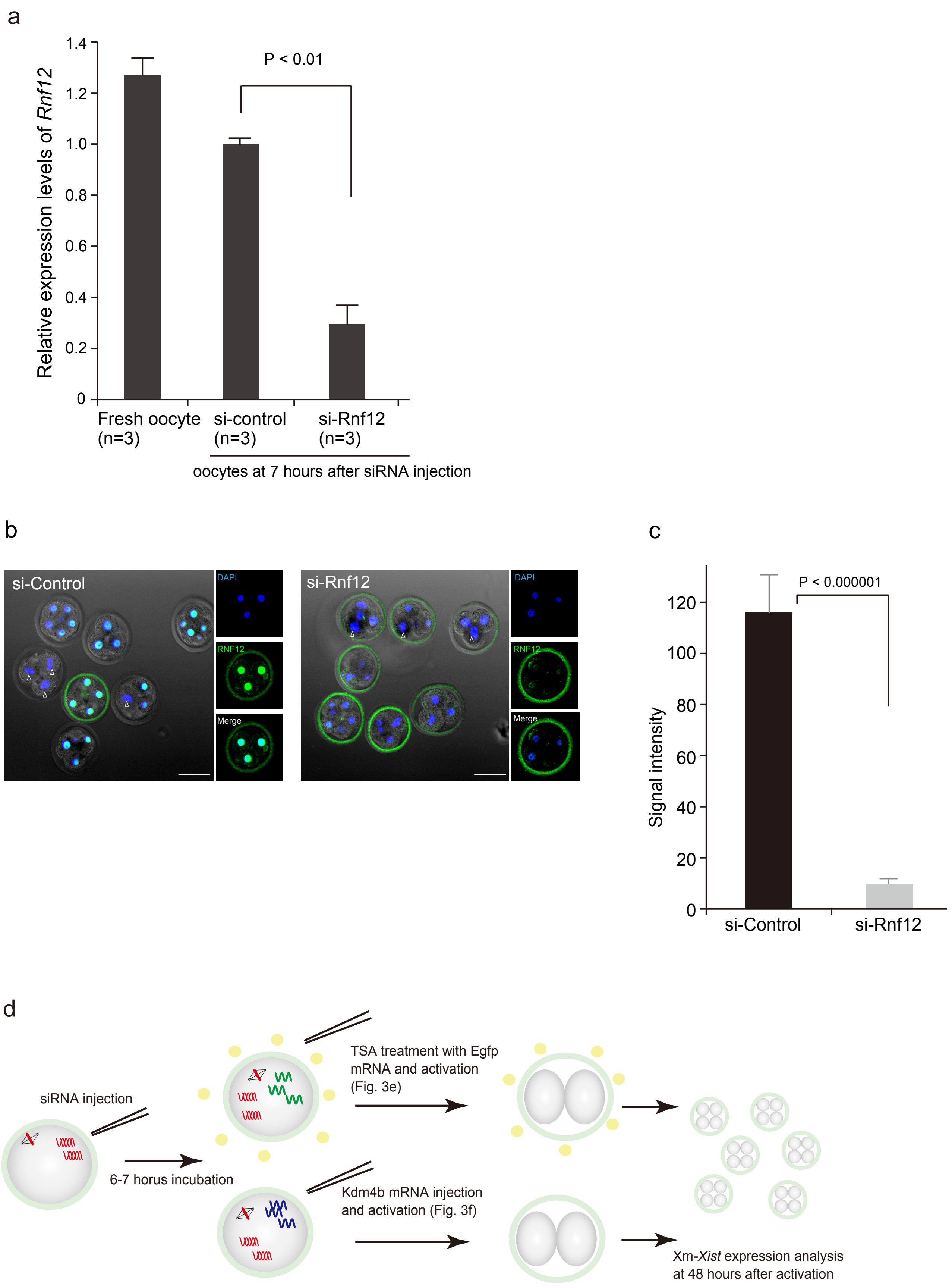

Generation of RNF12-depleted embryos using siRNA. (**a**) Analysis of the expression of maternal *Rnf12* in oocytes injected with siRNA targeting *Rnf12* (si-Rnf12) or control siRNA compared with that in uninjected fresh oocytes. (**b**) and (**c**) Immunofluorescence (IF) analysis of RNF12-depleted parthenogenetic embryos (PEs) at the 4-cell stage. Representative images of si-Rnf12- and control siRNA (si-Control)-injected embryos are shown. Scale bars = 50 μm. The delta symbol shows mitotic blastomeres that were omitted from quantification (b). Signal quantification of siRNA-treated embryos. Seven embryos were analysed in both groups (**c**). (**d**) Schema of *Kdm4b*- and *Egfp*+TSA-PEs derived from maternal/zygotic RNF12-depleted oocytes. The *P*-values were determined using Student's *t*-tests. Error bars indicate the mean ± SEM.

Supplemetary Fig. 7

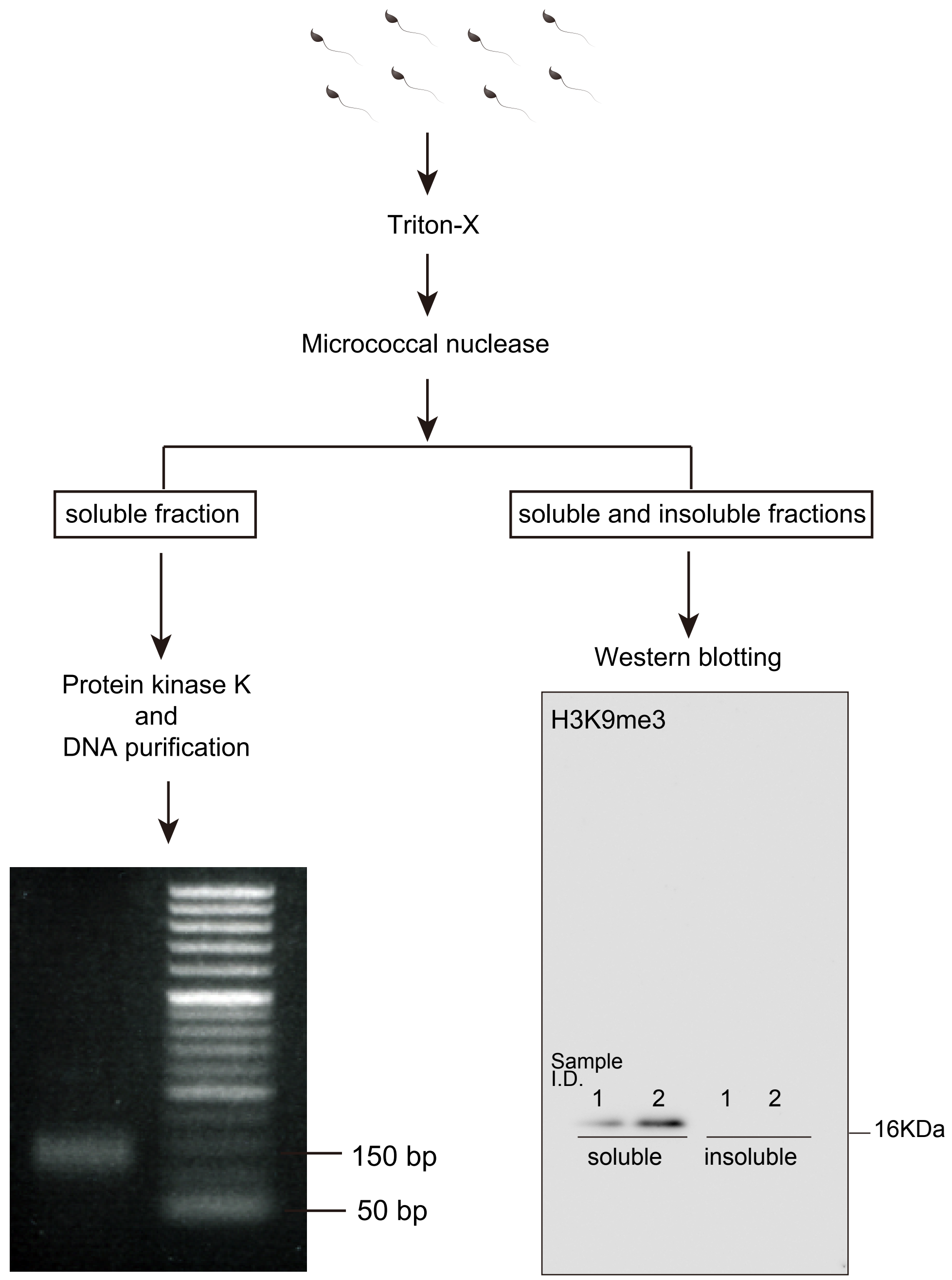

Scheme of recovery of sperm mononucleosomes. Electrophoresis of mononucleosomal DNA and western blotting analysis from each fraction used for the sperm ChIP-qPCR assay.

Supplementary Fig. 8

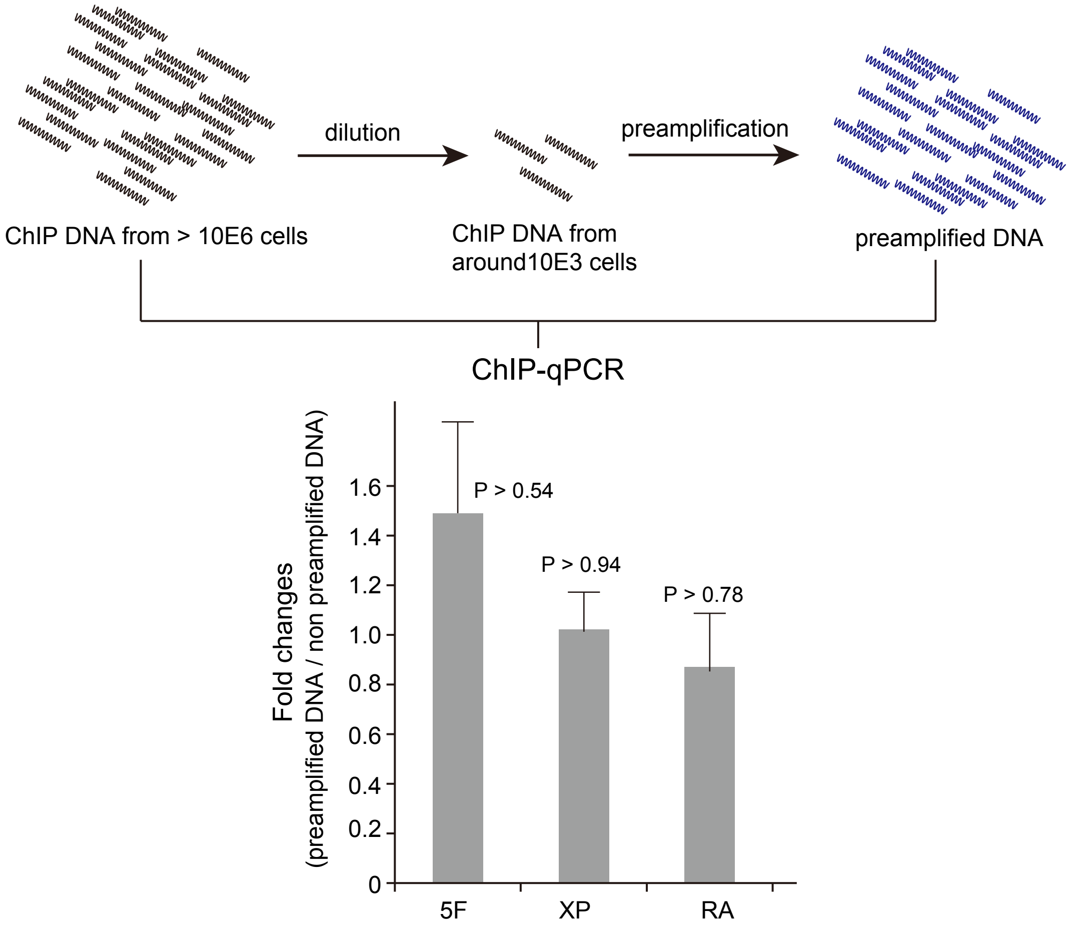

Comparison of ChIP-qPCR results from bulk DNA and pre-amplified DNA. Bulk ChIP DNA from embryonic stem (ES) cells were diluted and pre-amplified. ChIP-qPCR analysis was performed using bulk and pre-amplified DNA with a TaqMan probe targeting three *Xist* 5′ -regions. The *P*-values were determined using Student's *t*-tests. Error bars indicate the mean ± SEM.

Supplementary Fig. 9

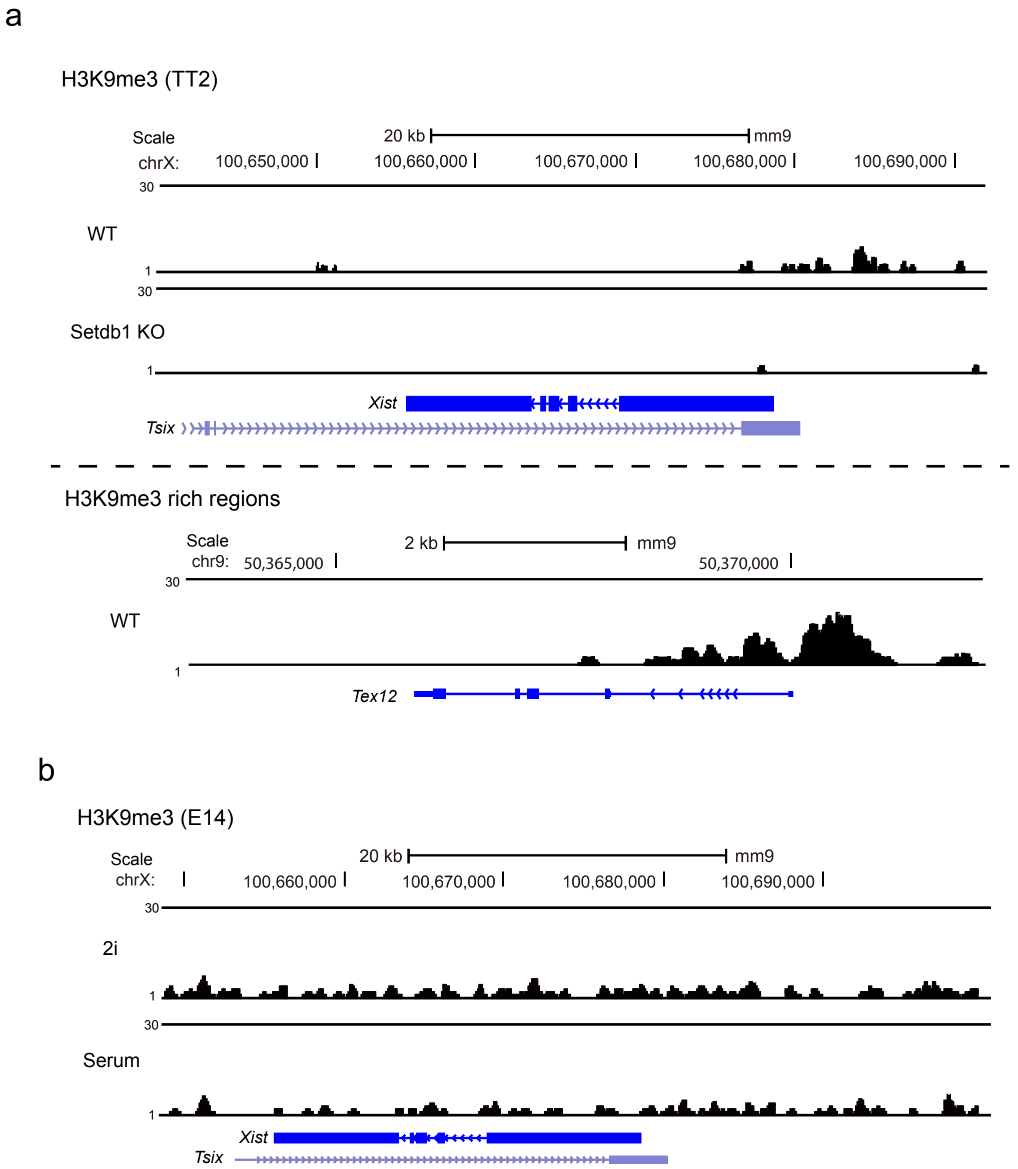

***In silico* analysis of H3K9me3 at the *Xist* locus in TT2 and E14 male ES cell lines.** ChIP-seq data for male TT2 and E14 ES cell lines acquired by Karimi *et al.*[30] (**a**) and Marks *et al.* [29] (**b**), respectively, were obtained from the Gene Expression Omnibus (GEO) database (http://www.ncbi.nlm.nih.gov/gds) under accession numbers GSE29413 and GSE23943, respectively. The data were visualised by importing into the Custom track of the University of California, Santa Cruz Genome Browser (http://genome.ucsc.edu/). The *Tex12* locus harbours an H3K9me3-rich region similar to the E14 line (data not shown).

Supplementary Fig. 10

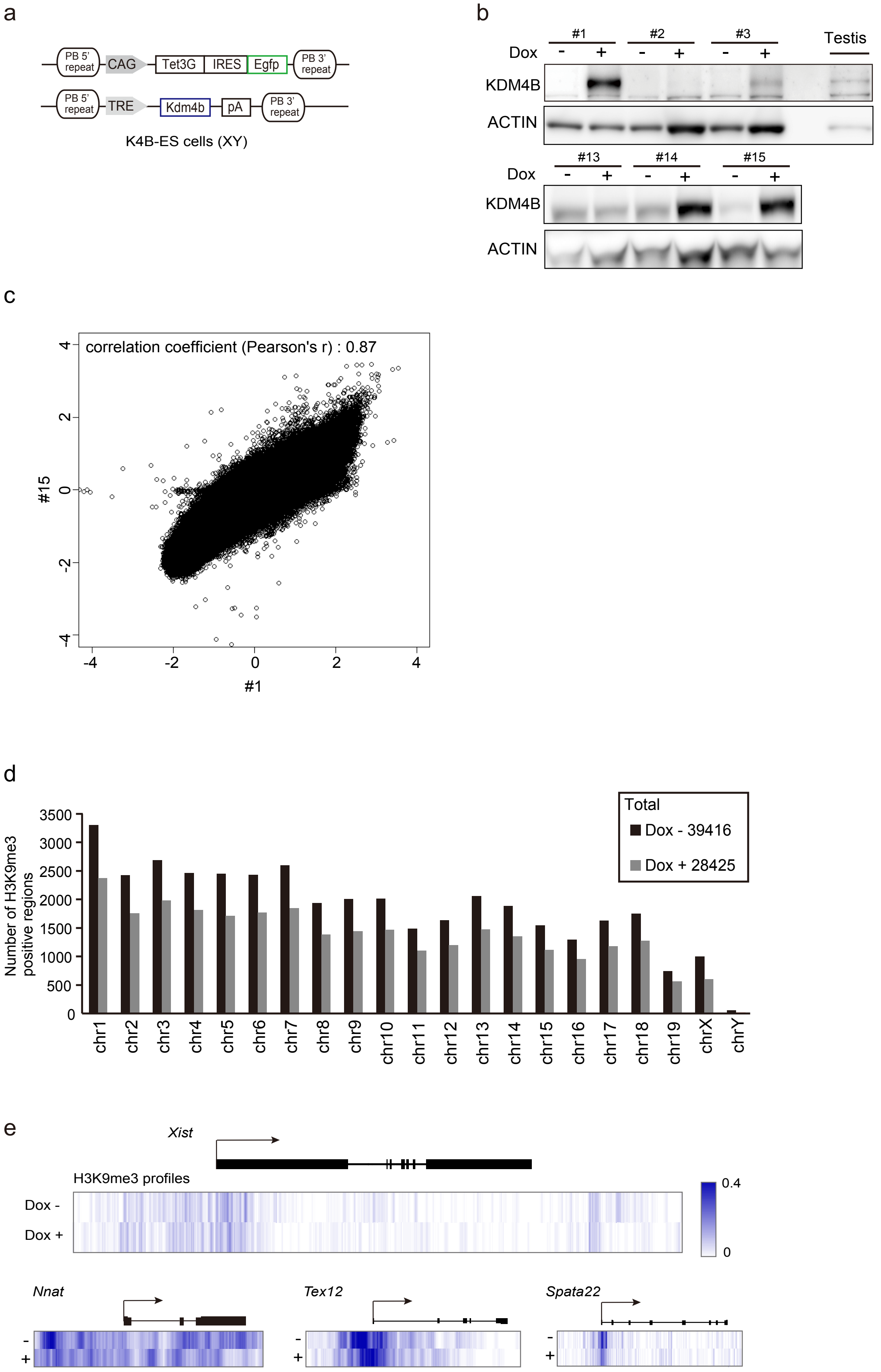

f

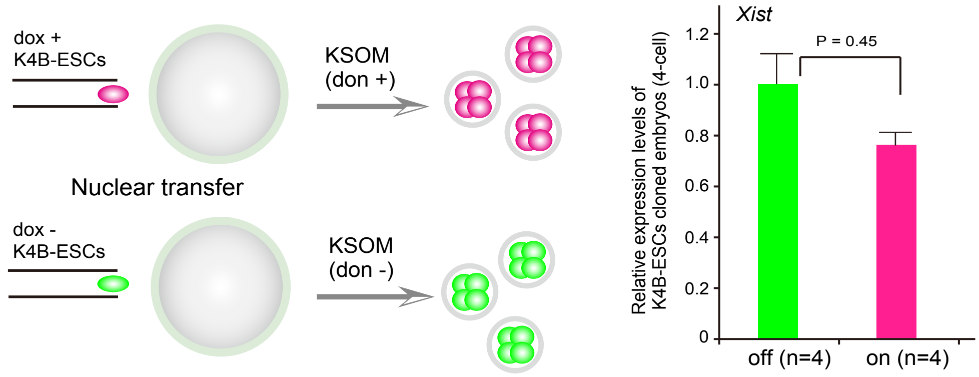
dox +
K4B-ESCs
KSOM
(don +)
Nuclear transfer
dox -
K4B-ESCs
KSOM
(don -)
Xist
P = 0.45
Relative expression levels of K4B-ESCs cloned embryos (4-cell)
1.2
1.0
0.8
0.6
0.4
0.2
0
off (n=4)
on (n=4)

g

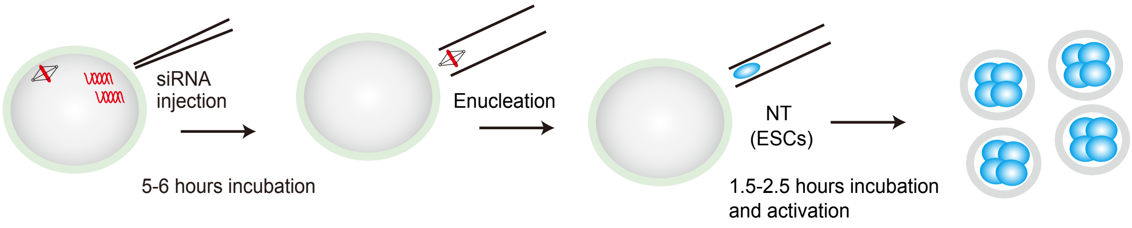
siRNA
injection
5-6 hours incubation
Enucleation
NT
(ESCs)
1.5-2.5 hours incubation
and activation

h

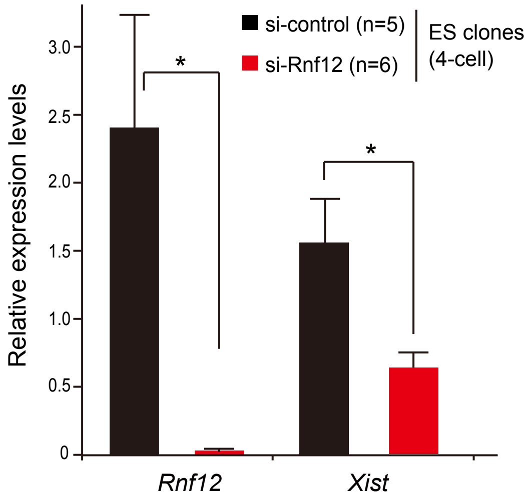
si-control (n=5)
si-Rnf12 (n=6)
ES clones
(4-cell)
Relative expression levels
3.0
2.5
2.0
1.5
1.0
0.5
0
*
*
Rnf12
Xist

Generation of KDM4B-inducible embryonic stem (ES) cells and RNF12 dependency of ES-cloned embryos. (**a**) Diagram of the PiggyBac doxycycline (Dox)-inducible expression vector used for introduction of *Kdm4b* into male ES cells (K4B-ES cells). (**b**) Western blot analysis of KDM4B expression after dox treatment for 2 days. (**c**) Scatter plots showing the relationship between two independent Dox-K4B-ES cell lines (#1 and #15). (**d**) H3K9me3-rich regions in the genomes of K4B-ES cells (#1). H3K9me3-positive regions in doxycycline on/off K4B-ES cell lines were identified using model-based analysis for ChIP-Seq (MACS). The numbers revealed a significantly enriched locus compared with the input data (false discovery rates < 1%, and elevated by a factor of greater than 5-fold). (**e**) H3K9me3 levels at *Xist* regions were relatively lower than those at known H3K9me3-rich regions (*Nnat*, *Tex12*, and *Spata22*)[30] in the #1 line. The heat maps show H3K9me3 levels (upper: dox$^-$, lower: dox$^+$). (**f**) Effects of H3K9me3 demethylation induced by ectopic expression of KDM4B on *Xist* expression in the NT embryos from K4B-ES cells (#1). *Xist* expression was determined using qPCR at the 4-cell stage in a pool of five cloned embryos. (**g**) Experimental scheme for generating *Rnf12*-knockdown cloned embryos. (**h**) qPCR analysis of *Xist* expression at the 4-cell stage of ES cell-cloned embryos using si-Rnf12-treated oocytes as recipients. A pool of five 4-cell embryos represents one biological replicate. Asterisks show P < 0.04. The P-values were determined using Student's t test. Error bars indicate the mean ± SEM.

Supplementary Fig. 11

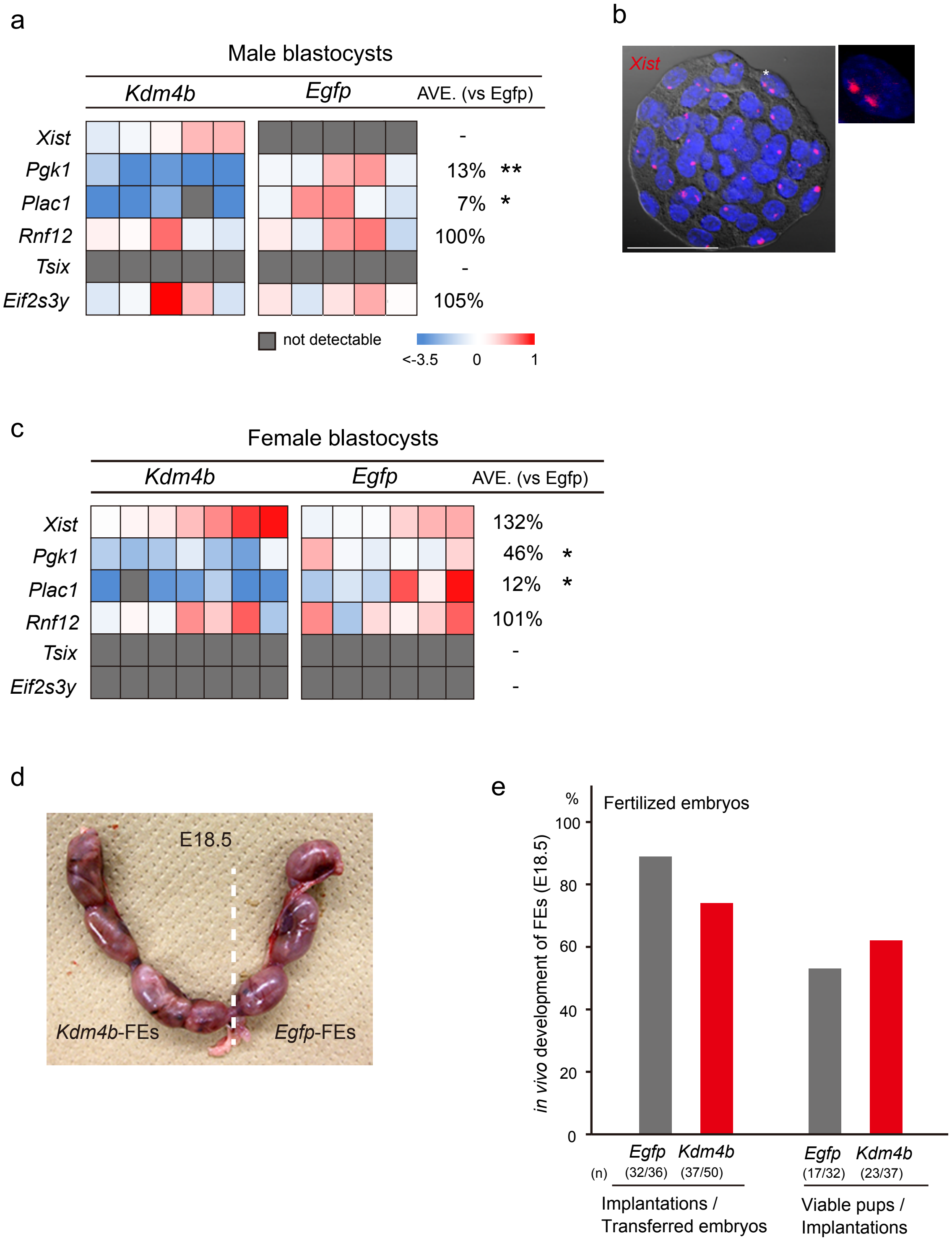

Effects of ectopic expression of Xm-*Xist* on developmental competency in FEs. (**a**) Expression of *Xist, Pgk1*, *Plac1*, *Rnf12, Tsix*, and *Eif2s3y* in individual male 96h blastocysts, as determined using qPCR. The genders of the embryos were determined by the detection of *Eif2s3y* located on the Y chromosome. The *P*-values were determined using Student's *t*-tests. Average expression levels are indicated by "Ave." and represent $**P < 0.001$ and $*P < 0.01$. The coloured bar scale is $\log_2$. (**b**) *Xist* FISH analysis of *Kdm4*b-FEs (female). The asterisks indicate cells with biallelic *Xist* expression. Scale bars = 50 µm. (**c**) qPCR analysis of X-linked gene expression in female embryos. The coloured bar scale and asterisks are the same as in (**a**). (**d**) Conceptus of *Kdm4b*- and *Egfp*-FEs. Representative images of the E18.5 uterus of pseudopregnant mice. *Kdm4b*-FEs (Xm-XCI) and *Egfp*-FEs were transferred into the left or right region of the uterus, respectively. (**e**) Full-term developmental rates. Five independent recipients were analysed, and the numbers of transferred embryos, implantations, and pups are shown in the graph.

Supplementary Fig. 12

a

Donor cell	No.of ET	No.of pups (%)
4-cell	47	15 (32.1)
8-cell	87	2 (2.3)
Morula	9	0
ICM	96	7 (7.3)
ES	196	14 (7.1)

b

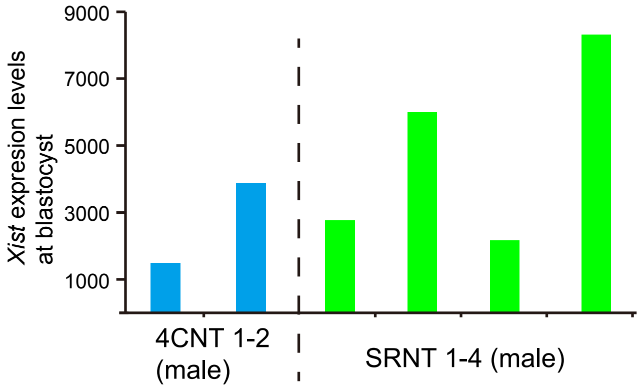

c

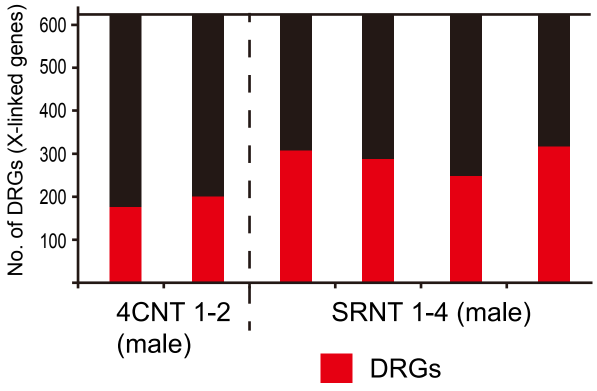

d

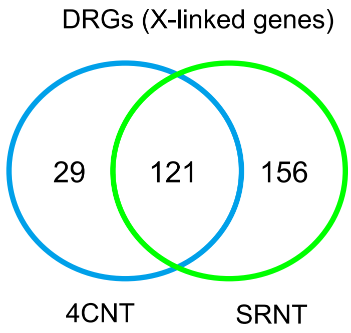

Association of developmental competency with XCI in cloned embryos. (**a**) Development of embryonic cloned embryos[31]. (**b**–**d**) Microarray analysis of 4-cell (4CNT) and Sertoli (SRNT)-cloned blastocysts using the previous reported data (GSE23181)[28]. (**b**) *Xist* expression states in cloned embryos. (**c**) X-linked genes (625) expressed in blastocysts were analysed. Genes repressed to less than 30% of the levels in IVF male embryos were identified as downregulated genes (DRGs). Red bars show DRGs. (**d**) Venn diagram of DRGs in cloned embryos, indicating that 80% of DRGs in 4CNT were the same as in SRNT. These results showed that high developmental competency was retained even when ectopic *Xist* was expressed and global XCI occurred.

Supplementary Fig. 13

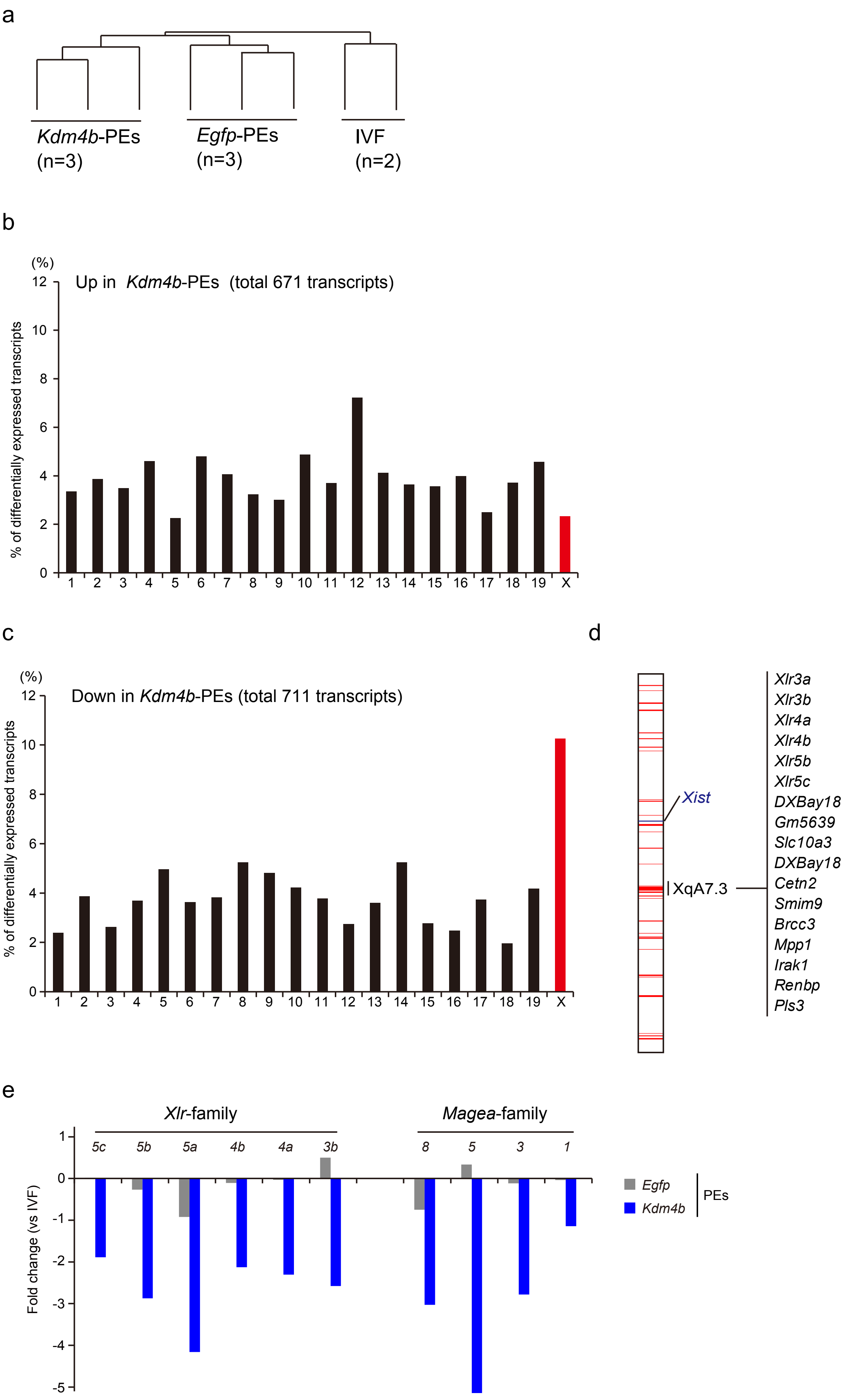

Transcriptome analysis of *Egfp*-PEs, *Kdm4b*-PEs, and IVF embryos at the blastocyst stage (120 h). (**a**) Unsupervised clustering analysis of gene expression. (**b**) and (**c**) Chromosome distribution of significantly differentially expressed transcripts between *Egfp*- and *Kdm4b*-PEs (Student's t test: P < 0.05, and more than 1.5-fold change). Genes upregulated (**b**) and downregulated (**c**) in *Kdm4b*-PEs. (**d**) Mapping of X-linked downregulated transcripts (DRTs) on the X chromosome. Twenty-one of 75 (28%) X-linked DRTs (17 genes) were located in the XqA7.3 region. (**e**) Fold-enrichment of *Xlr*- and *Magea*-family genes as determined by microarray data in *Egfp*- and *Kdm4b*-PEs compared to IVF embryos.

Supplementary Fig. 14

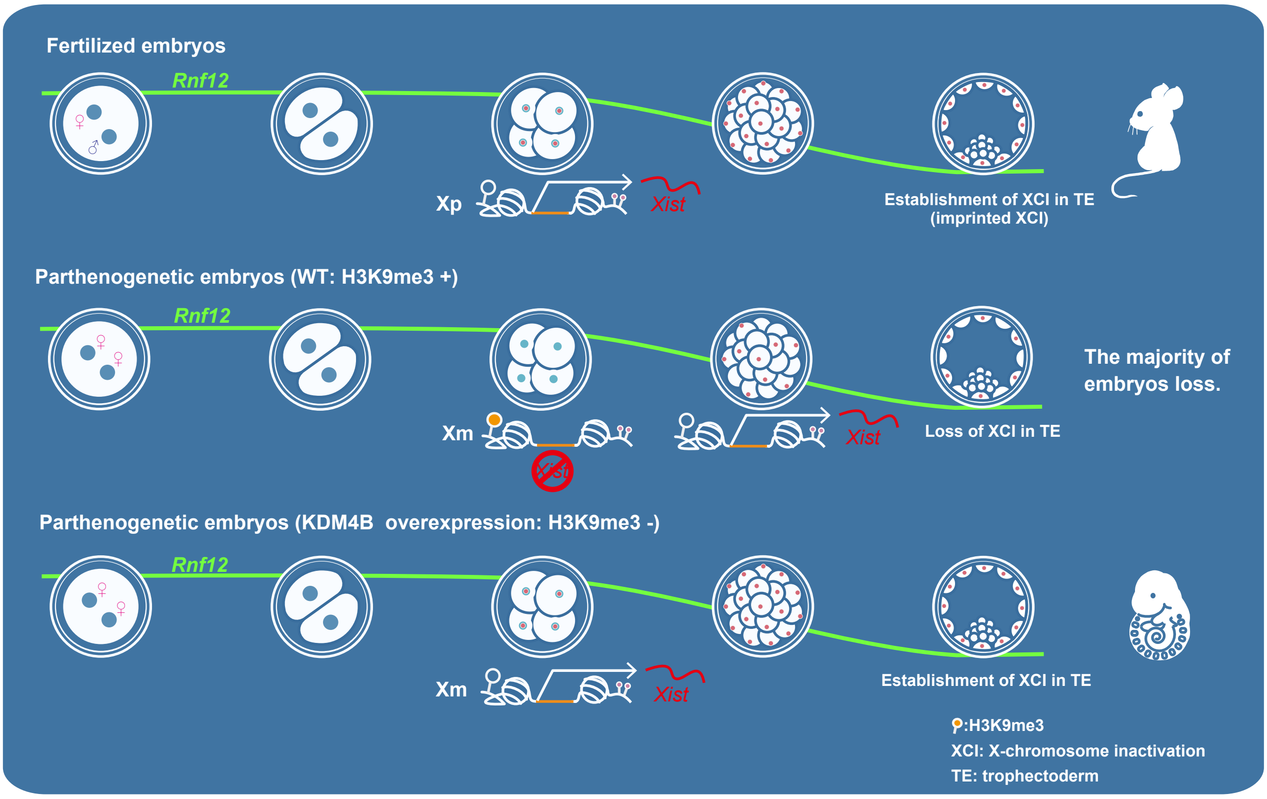

Summary of *Xist* regulation during pre-implantation phases and XCI impact on embryo development. Maternal-specific H3K9me3 prevents Xm-*Xist* activation by RNF12. Erasure of H3K9me3 at the *Xist* promoter region leads to Xm-*Xist* activation at the 4-cell stage and markedly improves developmental ability in PEs.

Table 1. *Xist* FISH analysis at the 4-cell stage.

PEs (n=emrbyos)	DAPI	cloud	pinpoint	biallelic cells	no signal cells	P-values (vs *Egfp*)
Egfp (12)	46	7	4	0	35	-
Kdm4b (16)	62	47	20	21	16	5.60E-11
Egfp +TSA (22)	84	31	16	11	47	0.005

The P-values were caluculated by Fisher’s exact test.

Table 2. *in vitro* development of PEs.

PEs	2-cell	4-cell	Morula	Blastocyst	% (Bl/2C)
Egfp	140	138	129	112	80.0%
Egfp +TSA	52	50	49	45	86.5%
Kdm4b	68	67	65	59	86.8%
Kdm4b +TSA	85	81	78	73	85.9%

Table 3. Immunofluorescence combined with *Xist* FISH analysis at the 96 h blastocysts.

Types		DAPI	cloud cells	ratio	p - values (vs Egfp)	biallelic cells	H3K27me3+ % (K27me3+ / cloud)
Egfp	PEs	720	261	36.3%	-	16	67.0%
Egfp +TSA		606	384	63.4%	5.46E-23	36	33.8%
kdm4b		690	502	72.8%	6.78E-44	62	41.4%
Kdm4b +TSA		973	729	74.9%	4.54E-58	100	22.1%
Female	FEs	364	333	91.5%	6.66E-75	0	75.4%

The P-values were caluculated by Fisher’s exact test.

Table 4. Immunofluorescence combined with *Xist* FISH at the 120 h blastocysts.

Types		DAPI	cloud	ratio	p - values (vs Egfp)	biallelic cells	H3K27me3+ % (K27me3+ / cloud)
Egfp	PEs	1619	462	28.5%	-	0	88.1%
Egfp +TSA		984	539	54.8%	2.93E-40	2	78.7%
Kdm4b		1323	838	63.3%	5.98E-81	2	91.8%
Kdm4b +TSA		1186	837	70.6%	1.66E-110	23	91.2%
Female	FEs	491	469	95.5%	4.50E-171	0	96.8%

The P-values were caluculated by Fisher’s exact test.

Table 5. *Xist* Fish analysis in *Rnf12* knockdown PEs at the morula stage.

Types		DAPI	cloud	pinpoint	no signal
si-control (n=12)	PEs	227	129	52	57
si-Rnf12 (n=12)		198	44	55	64

Table 6. *in vitro* development of *Kdm4b* mRNA injected fertilized embryos.

Types	2-cell	4-cell	Morula	Blastocyst	% (Bl/2C)
Kdm4b -FEs	64	61	61	56	87.5%
Egfp -FEs	26	26	24	22	84.6%

Table 7. Immunofluorescence combined with *Xist* FISH in the *Kdm4b*-PEs.

Types		ICM			TE		
		DAPI	*Xist*	H3K27me3	DAPI	*Xist*	H3K27me3
Egfp (n=13)	PEs	128	43	19	1058	340	302
Kdm4b (n=16)		156	66	17	1453	754	667

Late stage of blastocysts (120 h) were analyzed.

Table 8. *Xist* FISH analysis of *Rnf12* knockdown *Kdm4b*-PEs at the blastocyst stage.

Types		DAPI	cloud	pinpoint
si-control (n=12)	*Kdm4b*-PEs	763	396	90
si-Rnf12 (n=10)		552	55	290

Table 9. Primer/Probe sequences.

IVT template	*Kdm3a*	forward	CCTGCAGGTCTAATACGACTCACTATAGGGGCCACCATGG
		reverse	(120 x T)-AAGGTTTGCCCAAACTGGATTCACTG
	Kdm4b	forward	TAATACGACTCACTATAGGGCTCCACTTTGCTGCAACCATGGGGTCC
		reverse	(120 x T)-AGGAGTGGGCAGGATCTAGAAGGGTGCTCC
RT-PCR	*Kdm3a*	forward	TCTTGGGGATGTGGTGTTTATC
		reverse	TCCTGAGTAAGCCAGAAGCAGT
	Kdm4b	forward	AACCGCAATGGGCTCTACTAT
		reverse	AGTCTCTGCTCGTGATGCTCTCT
ChIP-qPCR	R1	forward	GGTGGACTTACCTTTCTTTCATTGTTT
		reverse	AAGAATGAAAAGGCAGGTAAGTAT
	R2	forward	ATGGCTGGAGCAAGCCGTTGCACG
		reverse	TAAAGGTCCAATAAGATGTCAGAA
	R3	forward	GGAACCAAGGAGCCATTTTGT
		reverse	CTTCTGCATTAGTTGGCGACC
	R4	forward	ATGAGGAGAGGAAAGGGTAGAAAT
		reverse	TAAAGGTCCAATAAGATGTCAGAA
	R5	forward	CCCTACCTGAACCACCTCAATAGT
		reverse	AGTTCCCTTTAGGCGTCCCAT
	H19	forward	AATCAACAAGGTCGGCTTACTCT
		reverse	ATCCGTTTTAGGACTGCGATGTA
	IAP	forward	GCTCCTGAAGATGTAAGCAATAAAG
		reverse	CTTCCTTGCGCCAGTCCCGAG
	Major sattellite	forward	GACGACTTGAAAAATGACGAAATC
		reverse	CATATTCCAGGTCCTTCAGTGTGC
	5'R	forward	AGATGAGGAGAGGAAAGGGTAGAAAT
		probe	FAM-CCTCACAAAATGGC-MGB
		reverse	AGAGAAAGACTAAGATGCCAATGACC
	XP	forward	GAGCAAGCCGTTGCACG
		probe	FAM-CTTTAACTGATCCGCGGCG-MGB
		reverse	ACCTAAAGGTCCAATAAGATGTCAGAA
	RA	forward	AAAAAAGAATGAAAAGGCAGGTAAGTAT
		probe	FAM-ACACACAGGTATCCGTGGC-MGB
		reverse	GGTGGACTTACCTTTCTTTCATTGTTT
	Gapdh	forward	CATCCAGGGACGTGCTGACT
		probe	FAM-CTGCCCTCGTGGACA-MGB
		reverse	TGTGTTCTCCCCTCACTGATCTC
TaqMan or quantitative probe	Gene	I.D.	
	Gapdh	Mm99999915_g1	
	Actb	Mm00607939_s1	
	Rnf12	Mm00488044_m1	
	Pgk1	Mm00435617_m1	
	Mecp2	Mm01193537_g1	
	Plac1	Mm00457647_m1	
	Tsix	Mm03455646_m1	
	Fmr1nb	Mm04178826_m1	
	Atrx	Mm00494196_m1	
	Uba1	Mm00493988_m1	
	Xist	Mm01232884_m1	
	Sfmbt2	Mm00616783_m1	
	Gnas	Mm01242435_m1	
	H13	Mm00468786_m1	
	Impact	Mm00492647_m1	

Xist targeting exons 6-7 (Figure 1f)	forward	CCCAAAGCAGCACAGAAAAC
	probe	FAM-ACCCGAGGATCAACATGCCTGAC-BHQ
	reverse	AGACACACCCACAATACACAC
